# Volatile sedation in critically ill adults undergoing mechanical ventilation: a meta-analysis of randomized controlled trials

**DOI:** 10.1186/s13054-025-05467-8

**Published:** 2025-06-05

**Authors:** Taihei Yamamoto, Yuki Kotani, Koya Akutagawa, Tomohisa Nagayama, Maho Tomimatsu, Mayuko Tonai, Toshiyuki Karumai, Yoshiro Hayashi

**Affiliations:** 1 Department of Intensive Care Medicine, Kameda Medical Center, 929 Higashi-cho, Kamogawa, 296-8602 Japan; 2 Emergency and Trauma Center, Kameda Medical Center, Kamogawa, Japan

**Keywords:** Systematic review, Meta-analysis, Volatile anesthetics, Mechanical ventilation, Sedation, Intensive care units

## Abstract

**Background:**

Volatile sedation has been associated with lung-protective effects, attenuation of inflammatory responses, and reduced organ dysfunction in critically ill patients. However, whether these potential benefits may translate into improvements in clinically relevant outcomes remains unclear. The primary aim of this meta-analysis of randomized controlled trials (RCTs) was to test the hypothesis that volatile sedation, compared to intravenous sedation, would reduce mortality in critically ill adults receiving mechanical ventilation.

**Methods:**

This study was registered in the PROSPERO database (CRD42023458064). We searched MEDLINE, Embase, and the Cochrane Library from inception until March 18, 2025, for RCTs comparing volatile sedatives with intravenous sedatives in critically ill patients undergoing invasive mechanical ventilation. The primary outcome was mortality at the longest follow-up. The secondary outcomes included duration of mechanical ventilation, length of hospital and intensive care unit (ICU) stay, hypotension, acute kidney injury, delirium, postoperative nausea and vomiting, atrial fibrillation, and time from sedative discontinuation to extubation. A random-effects Mantel–Haenszel meta-analysis was used for data synthesis. Trial sequential analysis (TSA) was performed to assess the robustness of the pooled data for the primary outcome.

**Results:**

We included 21 RCTs, comprising 2367 patients. Compared to intravenous sedation, volatile sedation may increase mortality at the longest follow-up (262/1107 vs. 218/1106; relative risk: 1.17; 95% confidence interval, 1.02 to 1.35; low certainty). However, TSA suggested a lack of definitive conclusion, as the required sample size was 12,080. No meaningful effects were observed on secondary outcomes, except for slightly shortened time from sedation termination to extubation (mean difference, − 90.62 min; 95% confidence interval, − 124.64 to − 56.60; low certainty),

**Conclusions:**

This meta-analysis of RCTs showed that, compared to intravenous sedation, volatile sedation may increase mortality among mechanically ventilated critically ill adults. Based on the current randomized evidence, its use in the ICU should be limited to carefully selected clinical scenarios. Further research is needed to identify patient populations that may benefit from this sedation strategy.

**Supplementary Information:**

The online version contains supplementary material available at 10.1186/s13054-025-05467-8.

## Background

Sedation forms an essential component of care for critically ill patients undergoing invasive mechanical ventilation, aimed at reducing discomfort, anxiety, and stress while improving ventilator synchronicity [1]. Intravenous (IV) sedation, typically using propofol, midazolam, or dexmedetomidine, has long been the standard approach in intensive care units (ICUs). However, these agents have their drawbacks, including reliance on hepatic and renal metabolism for elimination, potential drug interactions, and challenges in monitoring sedation depth. Given the association between benzodiazepines and delirium, current guidelines recommend propofol or dexmedetomidine as first-line agents [1]. Nonetheless, both carry inherent risks [1–4], highlighting the need for alternative sedation strategies.

Volatile sedation has recently gained interest in ICUs, facilitated by advancements in anesthetic delivery systems such as anesthetic-conserving devices. Although traditionally limited to the operating room, its ICU adoption has been driven by distinct pharmacokinetic advantages and increased demand during IV sedative shortages amid the coronavirus disease 2019 (COVID-19) pandemic [5–7]. Unlike IV sedatives, volatile anesthetic agents are predominantly eliminated via exhalation rather than hepatic or renal metabolism, potentially enabling faster emergence. Furthermore, continuous monitoring of exhaled anesthetic concentrations allows for real-time assessment of anesthetic depth and elimination.

Volatile agents may also mitigate ventilator-induced lung injury, attenuate inflammatory responses, and reduce organ dysfunction, all of which may contribute to improved clinical outcomes [8–10]. Previous systematic reviews have indicated that volatile sedation may facilitate earlier extubation than IV sedation in critically ill patients [11–13]. However, these analyses largely relied on small single-center randomized controlled trials (RCTs) [6, 14–17], limiting the generalizability of their findings. Moreover, none of them was adequately powered to investigate patient-centered outcomes like mortality, warranting the conduct of a multicenter, phase III clinical trial.

In this regard, a recent large-scale, multicenter RCT (Sevoflurane for Sedation in ARDS [SESAR] trial) compared inhaled sevoflurane sedation with propofol sedation in patients with acute respiratory distress syndrome (ARDS) [18]. The publication of such high-quality randomized evidence warranted an updated evaluation of volatile sedation in ICU settings. The primary aim of this study was to test a hypothesis that volatile sedation, compared to IV sedation, would reduce mortality in critically ill adult patients receiving invasive mechanical ventilation. 

## Methods

This systematic review and meta-analysis followed the Preferred Reporting Items for Systematic Reviews and Meta-Analyses (PRISMA) guidelines [19], and was registered in the PROSPERO register (CRD42023458064) on September 10, 2023 with modifications made on October 21, 2024, March 19, 2025, and April 24, 2025 (details are found in the Supplementary material). First, we chose to limit the eligibility to RCTs while excluding propensity score-matched observational studies. This decision aligns with the Cochrane collaboration, which discourages combining RCTs and non-randomized studies in a single meta-analysis due to substantial differences in risk of bias and confounding [20]. Second, we extended the literature search period. Since the ultimate purpose of conducting a systematic review and meta-analysis is to assess the totality of evidence regarding the research topic of interest, missing large and high-quality trials (i.e., SESAR trial in this case) does not meet the role of meta-analyses. Third, we designated mortality, instead of time to extubation, as the primary outcome. Initially, due to the limited number and size of available trials, we focused on time to extubation as a surrogate of arousal after sedation termination. However, the recent publication of a large-scale trial offered sufficient statistical power to evaluate the effects on more relevant outcomes, such as mortality. Furthermore, the SESAR trial reported a potential increased in mortality with volatile sedation, raising safety concerns and reinforcing the importance of investigation into mortality.

The review question was formulated using the PICO framework, defining the population as adult patients (> 16 years) receiving invasive mechanical ventilation and sedation in the ICU, the intervention as volatile sedative agents, the comparator as IV sedative agents, and the primary outcome as mortality at the longest follow-up. Only RCTs were included.

### Search strategy and selection criteria

A systematic search was conducted in MEDLINE, Embase, and the Cochrane Library for studies published up to March 18, 2025. Eligible studies were RCTs comparing volatile sedative agents with IV sedatives in critically ill patients. Studies were excluded if they were non-RCTs, pediatric studies, observational studies, case reports, case series, reviews, meta-analyses, letters, editorials, or had overlapping populations, or did not report pre-specified outcomes. No language restrictions were applied. After removing duplicates, two independent reviewers screened titles and abstracts. Full-text articles were assessed for inclusion independently, with discrepancies resolved by a third senior reviewer. The full search strategy is provided in the Supplemental Materials.

### Data collection and risk-of-bias assessment

Data were extracted by two independent investigators using a standardized collection form, including publication details, study design, patient population, interventions, comparators, and outcomes.

Risk-of-bias was independently assessed by two investigators using the Cochrane risk-of-bias tool for randomized trials, version 2 (RoB 2) [21]. The Grading of Recommendations Assessment, Development and Evaluation (GRADE) framework [22] was applied to evaluate the certainty of evidence. GRADEpro software [23] was used to generate profile tables. Publication bias and small-study effects were assessed using funnel plot analysis and Egger’s test.

### Outcomes

The primary outcome was mortality at the longest follow-up available. The secondary outcomes included duration of mechanical ventilation, length of hospital and ICU stay, hypotension, atrial fibrillation, acute kidney injury (AKI), delirium, postoperative nausea and vomiting (PONV), time from sedation termination to extubation. AKI and delirium were defined according to criteria specified in each study. We also assessed PaCO_2_ and pH as exploratory physiological outcomes in relation to volatile sedation delivery devices and their dead space volumes.

### Statistical analysis

A random-effects Mantel–Haenszel model was used for all analyses. Results were reported as mean difference (MD) for continuous data and relative risk (RR) for dichotomous data, both along with 95% confidence intervals (CIs). Statistical heterogeneity was assessed using Cochran’s Q test and quantified using the I^2^ statistic, with I^2^ > 50% indicating substantial heterogeneity. Statistical significance was set at *P* < 0.05.

For the primary outcome, we also conducted a Bayesian hierarchical random‐effects meta‐analysis on the RR scale using the *bayesmeta* package in R [24]. Study‐level log-RRs were calculated with a 0.5 continuity correction applied only to trials with a zero event in one arm (trials with zero event in both arms were retained without correction). We used a weakly informative prior for the overall log-RR, µ ~ Normal (0, 0.712^2^) so that 95% of its prior mass lies between RR = 0.25–4. For the between-study standard deviation (τ), we used an empirical heterogeneity prior based on the predictive distribution derived from hundreds of Cochrane meta-analyses that reported all-cause mortality [25]. After fitting the model, we computed the posterior probability of harm (RR > 1).

Pre-specified sensitivity analyses were conducted based on (1) type of volatile agent (sevoflurane, isoflurane, desflurane, or either of them within each study); (2) use of propofol as the comparator; (3) studies addressing patients with acute respiratory distress syndrome (ARDS); (4) surgical versus non-surgical settings; and (5) studies with overall low risk-of-bias according to RoB 2 assessment. Furthermore, to explore the potential sources of heterogeneity in treatment effects, post-hoc subgroup analyses were performed based on volatile anesthetic delivery device, COVID-19 status, and sedation duration (< or ≥ 24 h). These conventional meta-analyses were performed using RevMan Web (Version 8.10.0). Other analyses were conducted in R 4.2.1.

We also performed a trial sequential analysis (TSA) to investigate the robustness of the meta-analyzed results of mortality. We used the observed mortality rate in the control arm and assumed a 10% relative risk reduction with volatile sedation, with a diversity-adjusted required information size calculated using a two-sided alpha of 0.05 and a power of 80%. Moreover, TSA was conducted for length of ICU stay, AKI, and delirium, assuming an expected effect size of a 24-h reduction for ICU stay and a 20% relative risk reduction for AKI and delirium. TSA was performed using the TSA Viewer software (Version 0.9.5.10 Beta. Copenhagen Trial Unit, Centre for Clinical Intervention Research, Rigshospitalet, Copenhagen, Denmark).

Exploratory physiological outcomes were descriptively evaluated in relation to volatile sedation delivery devices and their dead spaces.

## Results

**Fig. 1:**
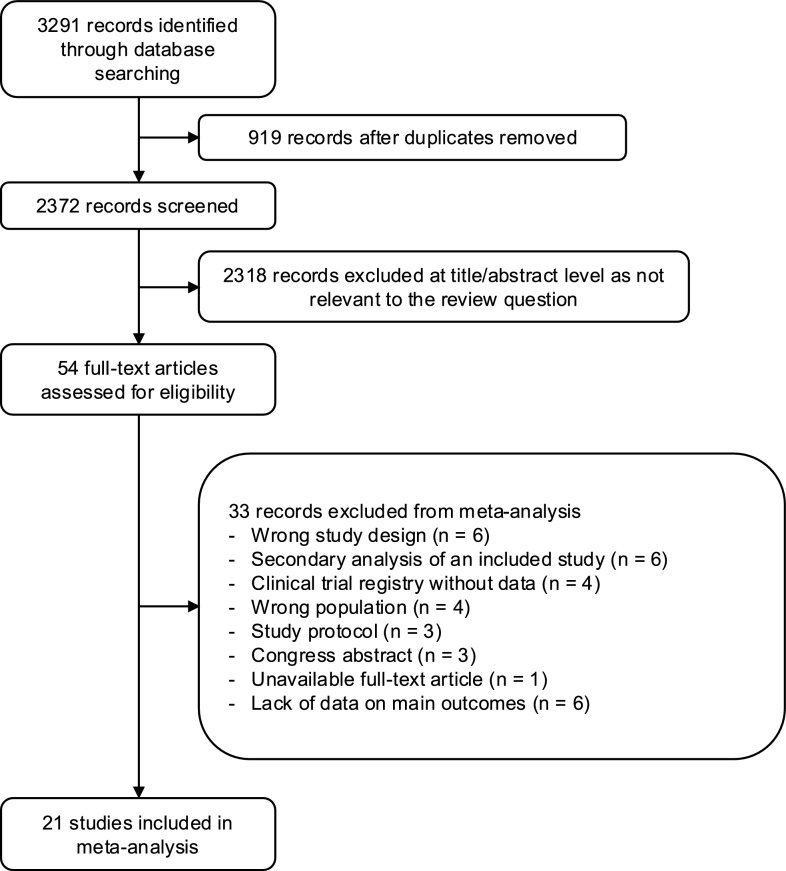
Flow chart of study selection

A total of 3219 studies were identified through database searches, of which 21 RCTs (2367 patients) met the eligibility criteria [6, 14–18, 26–41] (Fig. 1). Reasons for exclusion are detailed in Supplementary Table S1. The included studies were published between 1989 and 2025. Fourteen studies were conducted in surgical ICUs [14, 15, 17, 26–28, 30, 32, 35–37, 39–41], while seven were in non-surgical ICUs [6, 16, 18, 29, 31, 34, 38].

The volatile anesthetics used included sevoflurane (12 studies) [6, 15–18, 30, 32, 34–36, 38, 41], isoflurane (6 studies) [14, 26, 27, 29, 31, 40], desflurane (1 study) [28], and either sevoflurane or isoflurane (2 studies) [37, 39]. The comparators were propofol (13 studies) [6, 14, 15, 17, 18, 28, 30, 32, 35–37, 39, 41], midazolam (5 studies) [26, 27, 29, 31, 38], and multiple IV agents, including dexmedetomidine and ketamine (3 studies) [16, 34, 40].

Study characteristics are summarized in Table 1. Risk-of-bias assessment based on RoB 2 showed that seven RCTs had a low risk-of-bias, ten had some concerns, and four were at high risk-of-bias (Supplemental Table S2).

**Table 1:** Characteristics of included studies

First author, year	No. of patients	No. of centers	Country	Patients	Volatile sedation	Intravenous sedation
Kong KL, 1989 [26]	60	1	UK	Patients needed mechanical ventilation for longer than 12 h	Isoflurane End tidal 0.1–0.6%	Midazolam
Spencer EM, 1992 [27]	60	1	UK	Mechanically ventilated patients expected to require sedation for longer than 24 h	Isoflurane End tidal 0.1–0.6%	Midazolam
Meiser A, 2003 [14]	56	1	Germany	Postoperative major surgery patients	Desflurane End tidal 0.3 & plus steps of up to 0.5%	Propofol
Sackey PV, 2004 [29]	40	1	Sweden	Mechanically ventilated patients expected to require sedation for longer than 12 h	Isoflurane End tidal 0.5%	Midazolam
Rohm KD, 2008 [30]	70	1	Germany	Postoperative cardiac surgery patients	Sevoflurane End tidal 0.5–1.0%	Propofol
Sackey PV, 2008 [31]	40	1	Sweden	Mechanically ventilated patients expected to require sedation for longer than 12 h	Isoflurane End tidal 0.5%	Midazolam
Rohm KD, 2009 [32]	125	1	Germany	Postoperative major surgery patients (including cardiac surgery)	Sevoflurane End tidal 0.5–1.0%	Propofol
Mesnil M, 2011 [34]	47	1	France	Mechanically ventilated patients expected to require sedation for longer than 24 h	Sevoflurane End tidal 0.5%	Midazolam or propofol
Hellstrom J, 2012 [35]	99	1	Sweden	Postoperative cardiac surgery patients	Sevoflurane End tidal 0.5–1.0%	Propofol
Soro M, 2012 [36]	73	1	Spain	Postoperative cardiac surgery patients	Sevoflurane End tidal 0.5–1.0%	Propofol
Jerath A, 2015 [37]	141	1	Canada	Postoperative cardiac surgery patients	Sevoflurane, Isoflurane MAC 0.1–0.3	Propofol
Jabaudon M, 2017 [38]	50	1	France	Patients with moderate-to-severe ARDS within 24 h of onset	Sevoflurane Started at 6 ml/h and adapted every 15 min to reach targeted BIS value	Midazolam
Wascowicz M, 2018 [39]	127	1	Canada	Postoperative cardiac surgery patients	Sevoflurane or Isoflurane MAC 0.6–2.0	Propofol
Jerath A, 2020 [40]	60	2	Canada	Mechanically ventilated patients expected to require sedation for longer than 48 h	Isoflurane NA	Midazolam or propofol
Guinot PG, 2020 [41]	81	1	France	Postoperative cardiac surgery patients	Sevoflurane NA	Propofol
Meiser A, 2021 [14]	301	24	Germany and Slovenia	Mechanically ventilated patients expected to require sedation for longer than 24 h	Isoflurane MAC 0.5 ± 0.2	Propofol
Soukup J, 2023 [15]	79	1	Germany	Mechanically ventilated patients expected to require sedation for longer than 48 h	Sevoflurane End tidal 0.9–1.1%	Propofol
Martínez-Castro S, 2023 [6]	17	4	Spain	Patients with ARDS due to COVID-19	Sevoflurane NA	Propofol
Beck-Schimme B, 2024 [16]	60	4	Switzerland	Patients with severe COVID-19 related lung injury	Sevoflurane End tidal 1.2 ± 0.4	Midazolam or propofol or dexmedetomidine
Flinspach AN, 2024 [17]	94	1	Germany	Postoperative cardiac surgery patients	Sevoflurane MAC 0.7 ± 0.1	Propofol
Jabaudon M, 2025 [18]	687	37	France	Patients with early moderate to severe ARDS	Sevoflurane NA	Propofol

*MAC* minimum alveolar concentration; *ARDS* acute respiratory distress syndrome; COVID-19, coronavirus infectious disease 2019; *TTM* targeted temperature management; *VA-ECMO* veno arterial extracorporeal membrane oxygenation; *UK* United Kingdom; *NA* not applicable

### Primary outcome

**Fig. 2:**
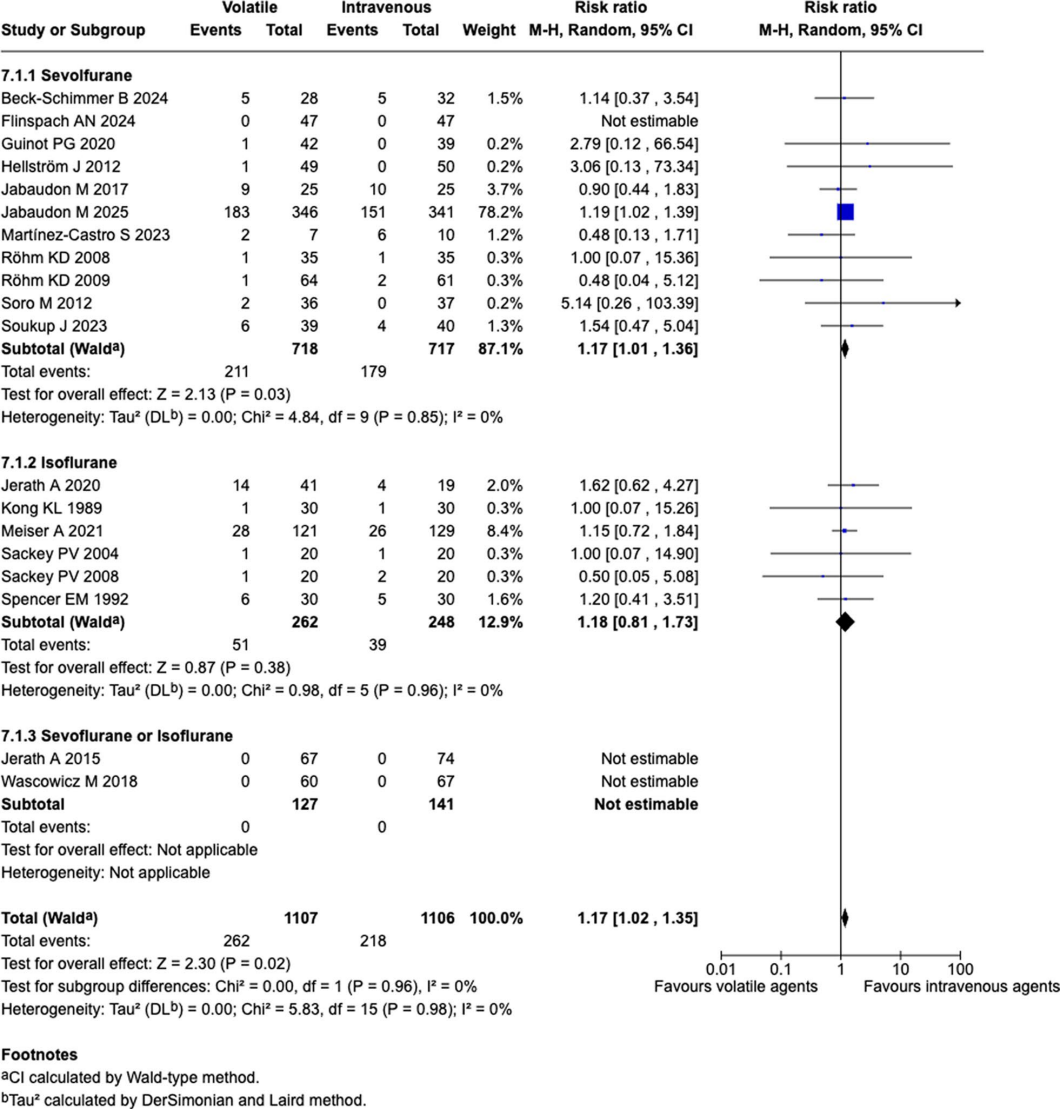
Forest plot for mortality at the longest follow-up using the frequentist analysis

**Fig. 3:**
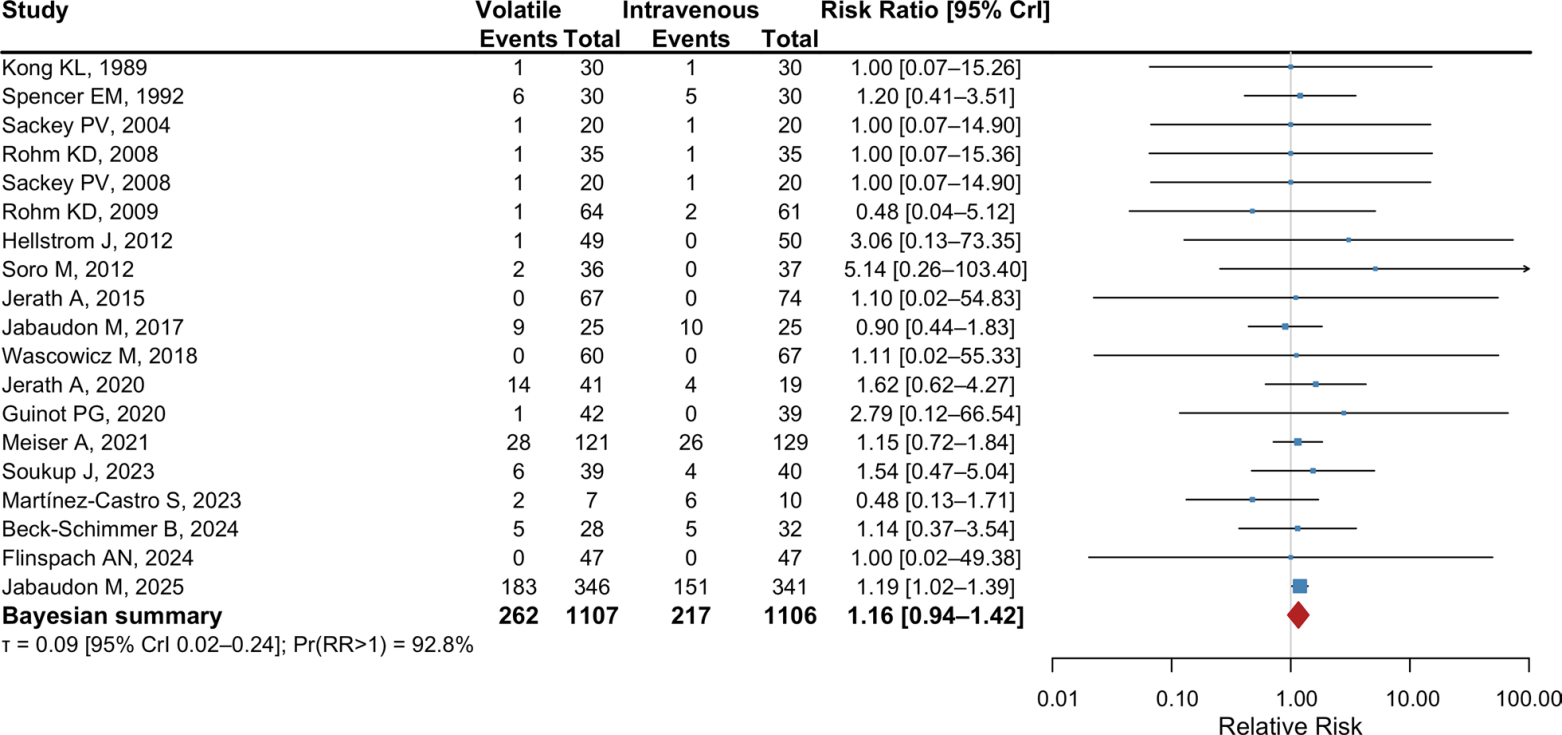
Forest plot for mortality at the longest follow-up using the Bayesian analysis. CrI, credible interval

Compared to IV sedation, volatile sedation may increase mortality at the longest follow-up available (19 studies; 262/1107 vs. 218/1106; RR, 1.17; 95% CI, 1.02 to 1.35; *P* = 0.02; I^2^ = 0%; Tau^2^ = 0; low certainty) (Fig. 2 and Table 2). A visual inspection of the funnel plot revealed a symmetrical distribution of effect sizes around the pooled estimate, with no studies falling outside the pseudo 95% confidence limits, suggesting no evidence of publication bias (Supplemental Figure S1). Egger’s test showed no significant evidence of small-study effects (bias coefficient = − 0.064 ± 0.186; *P* = 0.73). When we applied a Bayesian hierarchical random-effects model, the probability that volatile sedation increased mortality was 92.8% (RR, 1.16; 95% credible interval, 0.94 to 1.42; Fig. 3). Between-study heterogeneity was low (posterior standard deviation τ = 0.09; 95% credible interval, 0.02 to 0.24).

**Table 2:** Effects of volatile sedative on mortality at the longest follow-up

Group	No. of studies	Volatile sedation	Intravenous sedation	Relative risk	95% CI	*P* value	I^2^
Overall	19	262/1107	218/1106	1.17	1.02 to 1.35	0.02	0%
Sevoflurane	11	211/718	179/717	1.17	1.01 to 1.36	0.03	0%
Isoflurane	6	51/262	39/248	1.18	0.81 to 1.73	0.38	0%
Either sevoflurane or isoflurane	2	0/127	0/141	NA	NA	NA	NA
Propofol as comparator	12	224/902	191/922	1.18	1.02 to 1.36	0.03	0%
ARDS	4	199/412	172/410	1.16	1.00 to 1.35	0.05	0%
Surgical settings	13	61/661	43/658	1.27	0.89 to 1.81	0.19	0%
Non-surgical settings	6	201/446	175/448	1.16	1.00 to 1.35	0.05	0%
Low risk-of-bias studies	7	241/661	195/640	1.19	1.03 to 1.37	0.02	0%
Anesthetic conserving device	14	249/930	207/927	1.17	1.02 to 1.35	0.02	0%
Electronic anesthetic delivery system	1	1/42	0/39	2.79	0.12 to 66.54	0.53	NA
Anesthetic vaporizer	2	7/60	6/60	1.17	0.43 to 3.18	0.76	0%
COVID-19	3	190/381	162/383	1.18	1.01 to 1.37	0.04	0%
Without COVID-19	16	72/726	56/723	1.16	0.85 to 1.59	0.35	0%
Shorter sedation duration	4	4/178	4/176	0.96	0.25 to 3.73	0.96	0%
Longer sedation duration	5	232/558	187/539	1.20	1.04 to 1.38	0.01	0%

*ARDS* acute respiratory distress syndrome; *NA* not applicable

Subgroup analysis based on the volatile agent type showed that the overall result was mainly driven by sevoflurane studies; however, the point estimate was similar in isoflurane studies (Fig. 2). Although most sensitivity analyses, including those restricted to studies of surgical patients and to low risk-of-bias studies, were consistent with the primary analysis (Supplemental Figures S2–7), a subgroup of a shorter sedation duration (< 24 h) found a point estimate of RR < 1 (RR, 0.96; 95% CI, 0.25 to 3.73; Supplemental Fig. 8).

**Fig. 4:**
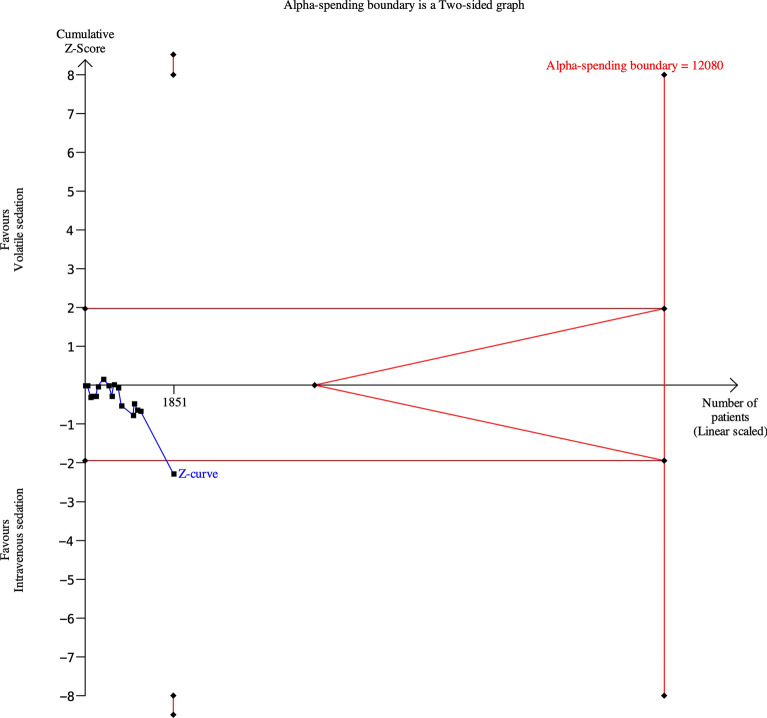
Trial sequential analysis for mortality at the longest follow-up. Alpha error = 5%, power = 80%, relative risk decrease = 10%, diversity = 0%

Figure 4 shows the TSA result. Although the cumulative Z-curve crossed the conventional significance boundary, it did not cross the monitoring boundaries and the accrued information size (i.e., 1851 patients) remained well below the required information size of 12,080. Thus, the current evidence is insufficient to draw a definitive conclusion, indicating the need for further adequately powered RCTs.

The timepoints for mortality assessment ranged from 24 h to 90 days in across 11 studies [6, 14, 16, 18, 26, 29, 31, 35, 38, 41, 42], with a median of 30 days and an interquartile range of 5.5 to 30 days. ICU mortality was reported in one study [17] and hospital mortality was reported in seven studies [15, 30, 32, 36, 39, 40, 43] (Supplemental Table S3). GRADE evaluation is reported in Supplemental Table S4.

### Secondary outcomes

The pooled results of the secondary outcomes are summarized in Table 3.

**Table 3:** Effects of volatile sedative on secondary outcomes

Outcome	No. of studies	Volatile sedation	Control	Relative risk	Mean difference	95% CI	*P* value	I^2^
Duration of mechanical ventilation, hour	8				− 1.46	− 4.92 to 2.00	0.41	53%
Length of hospital stay, day	11				− 0.37	− 0.93 to 0.18	0.18	19%
Length of ICU stay, hour	15				1.02	− 4.89 to 6.94	0.73	77%
Hypotension	10	128/531	134/561	1.01		0.75 to 1.36	0.95	43%
Atrial fibrillation	7	67/749	59/757	1.15		0.74 to 1.79	0.53	31%
Acute kidney injury	8	284/728	237/704	1.14		0.67 to 1.93	0.62	60%
Delirium	7	43/357	42/356	1.04		0.72 to 1.50	0.85	0%
Postoperative nausea and vomiting	8	39/478	31/465	1.29		0.82 to 2.01	0.27	0%
Time from sedation termination to extubation, minute	13				− 90.62	− 124.64 to -56.60	< 0.0001	90%

*ICU*, intensive care unit; *CI*, confidence interval

Volatile sedation likely has no effect on the ventilation duration (8 studies; MD, − 1.46 h; 95% CI, − 4.92 to 2.00; *P* = 0.41; I^2^ = 53%; moderate certainty) (Supplemental Figure S9).

Volatile sedation probably has no effect on length of hospital stay (11 studies, MD, − 0.37 days; 95% CI, − 0.93 to 0.18; *P* = 0.18; I^2^ = 19%; moderate certainty; Supplemental Figure S10). It may result in little to no difference in length of ICU stay (15 studies, MD, 1.02 h; 95% CI, − 4.89 to 6.94; *P* = 0.73; I^2^ = 77%; low certainty; Supplemental Figure S11) or in the incidence of hypotension (10 studies, RR, 1.01; 95% CI, 0.75 to 1.36; *P* = 0.95; low certainty; I^2^ = 43%; Supplemental Figure S12). The TSA indicated that the cumulative evidence is sufficient to rule out a clinically meaningful reduction in length of ICU stay with volatile sedation under the predefined assumptions (Supplementary Figure S13).

The evidence is very uncertain regarding the effect of volatile sedation on atrial fibrillation (7 studies, RR, 1.15; 95% CI, 0.74 to 1.79; *P* = 0.53; I^2^ = 31%; Supplemental Figure S14), AKI (8 studies, RR, 1.14; 95% CI, 0.67 to 1.93; *P* = 0.62; I^2^ = 60%; Supplemental Figure S15), delirium (7 studies, RR, 1.04; 95% CI, 0.72 to 1.50; *P* = 0.85; I^2^ = 0%; Tau^2^ = 0; Supplemental Figure S16), and PONV (8 studies, RR, 1.29; 95% CI, 0.82 to 2.01; *P* = 0.27; I^2^ = 0%; Tau^2^ = 0; Supplemental Figure S17). The TSA suggested the lack of evidence to draw a conclusion for the association between volatile sedation and AKI and delirium (Supplementary Figure S18 and Supplementary Figure S19).

Volatile sedation may slightly reduce the time from sedation termination to extubation compared to IV sedation (MD, − 91 min; 95% CI, − 125 to − 57; *P* < 0.001; low certainty; I^2^ = 90%; Supplemental Figure S20).

Most prespecified and post-hoc sensitivity analyses of secondary outcomes, including those based on volatile sedative agent (i.e., sevoflurane, isoflurane, or desflurane), were consistent with the main analyses (Supplementary Table S5 and Supplementary Table S6). Significant interactions were observed for the following outcomes: duration of mechanical ventilation based on COVID-19 status (P for interaction = 0.01), length of ICU stay based on sedation duration (P for interaction = 0.04), and time to extubation based on surgical versus non-surgical studies (P for interaction = 0.01).

### Exploratory physiological outcomes

In four studies, PaCO_2_ and pH levels were reported [6, 14, 18, 38]. Only one study using anesthetic conserving device with a dead space of 100 mL observed a sizable increase in PaCO_2_ and pH levels with volatile sedation [6] (Supplementary Table S7).

## Discussion

### Key findings

Based on the 21 RCTs totaling 2367 patients identified through our comprehensive literature search, we compared volatile sedation with IV sedation in critically ill adult patients undergoing invasive mechanical ventilation. This meta-analysis found a higher risk of mortality with volatile sedation than IV sedation, which was consistent across different volatile agent types and patient subgroups. No significant differences in secondary outcomes were observed except for a slightly shortened time from sedation termination to extubation with volatile sedation.

### Comparison with previous literature

Unlike previous studies with inconclusive mortality findings [11–13], this meta-analysis is the first to demonstrate an increased risk of death associated with volatile sedation in mechanically ventilated critically ill patients. This result contrasts with growing clinical data suggesting potential benefits of volatile sedation, particularly in terms of lung protection [38, 44, 45]. However, most earlier studies had fundamental limitations such as being single-center with small sample size, which precluded assessing clinically relevant outcomes like mortality.

The increased mortality observed in this meta-analysis was largely driven by the recent SESAR trial, which compared sevoflurane with propofol in patients with severe ARDS [18]. However, our sensitivity analyses excluding the SESAR trial (e.g., focusing on surgical patients or isoflurane studies) yielded a similar point estimate, suggesting a potential harm of volatile sedation across different agent types and patient populations. Nonetheless, the TSA indicated that the current body of evidence remains insufficient to confirm or refute a detrimental effect of volatile sedation on mortality, warranting further evaluation in future RCTs.

Propofol was the most common comparator in the included studies, and a sensitivity analysis limited to those studies yielded similar results, further suggesting that volatile sedation may be harmful compared to propofol. In contrast to our findings, intraoperative volatile anesthesia, compared to propofol-based anesthesia, has been associated with improved outcomes in cardiac surgery [3]. The much longer duration of administration in the ICU (days vs. hours in the operating theater) could have contributed to these contrasting results [46], although the underlying mechanisms remain uncertain.

Sevoflurane has been considered potentially nephrotoxic due to the formation of Compound A, a degradation product which may cause proximal tubular necrosis in a dose-dependent manner in animal models [47]. However, this theoretical risk has not been confirmed in clinical studies involving critically ill patients [48, 49]. In our meta-analysis, the point estimate favored IV sedation over sevoflurane, mainly driven by the SESAR trial [18]. Although prolonged use of sevoflurane may have played a role, the underlying mechanisms remain uncertain.

Previous meta-analyses have suggested that volatile sedation facilitate earlier extubation compared to IV sedation [11, 12, 50, 51], which is consistent with our result. This reinforces the notion that volatile anesthetics enable faster recovery due to their pharmacokinetic properties. Unlike IV sedatives, which rely on hepatic and renal metabolism and accumulate in patients with severe hepatic or renal dysfunction, volatile anesthetics are eliminated via exhalation, allowing for more predictable drug clearance and titration [52, 53].

### Implications for clinical practice and future research

The increased risk of mortality shown in our review implies that the routine use of volatile sedation should be avoided in mechanically ventilated ICU patients who require sedation. Importantly, this harmful effect was consistent across different volatile anesthetic agents and clinical scenarios assessed in our analysis.

However, the included studies did not address certain patient populations that might benefit from volatile sedation. For instance, no RCTs have examined its use with severe asthma. Although sevoflurane is widely used for this indication, a recent systematic review highlighted the lack of randomized evidence in this setting [54]. Given sevoflurane’s bronchodilatory properties and the pathophysiology of this disease, prospective evaluation is needed to determine whether sevoflurane, either as a primary sedative agent or in combination with IV sedatives, improves clinical outcomes in this specific scenario. Additionally, the ability to estimate anesthetic depth and residual drug effects through end-tidal volatile agent concentrations, along with the relatively rapid elimination of these agents via exhalation, may facilitate neurological prognostication in patients with conditions such as post-cardiac arrest syndrome.

Other potential indications for volatile sedation include cases where target sedation level cannot attained despite maximal IV sedation (e.g., refractory status epilepticus [55]), and situations where IV sedative shortages limit treatment options. Given the lack of robust evidence supporting its use, clinicians should carefully weigh the potential risks and benefits when considering volatile sedation in these selected cases.

The exact mechanisms underlying the worse outcome remain uncertain, warranting further mechanistic studies using biochemical data. Moreover, the TSA indicated that a definitive conclusion could not be drawn regarding the association between volatile sedation and increased mortality. Thus, the safety of using volatile sedation in the ICU needs further evaluation within the confines of clinical research.

Different volatile sedative agents may have varying effects on outcomes such as mortality and AKI. In this meta-analysis, sevoflurane was associated with increased mortality risks, whereas isoflurane did not show a statistically significant effect. For AKI, the point estimates suggested a lower risk with IV sedation compared to sevoflurane, but a potential benefit of isoflurane over IV sedation. Additionally, the duration of sedation varied across studies, ranging from several hours to seven days. Of note, a subgroup analysis based on sedation duration revealed that studies using a shorter duration found no difference in mortality, while studies using a longer sedation duration identified an increased mortality risk with volatile sedation. Similarly, a significant interaction was observed for length of ICU stay based on sedation duration. These variations in agent type and sedation duration may contribute to heterogeneity in treatment effects, which ongoing clinical trials (e.g., NCT05327296, NCT05312385, NCT04341350, NCT04415060) may help clarify.

As with other interventions delivered to critically ill patients, a one-size-fits-all approach does not apply to sedation in the ICU. Personalized sedation management, considering clinical course, patient characteristics, and sedative properties, should be prioritized in future research [56]. Although previous studies, including this meta-analysis, compared different sedatives as the primary agent, a multimodal sedation strategy incorporating volatile anesthetics might play a role. Such an approach has the potential to spare IV sedatives, mitigate adverse effects of individual agents, and avoid drug shortages as we experienced during the COVID-19 pandemic [5–7].

### Strengths and limitations

This review has several strengths. First, our comprehensive literature review provides the most up-to-date randomized evidence, which allowed sufficient statistical power to assess the effect of volatile sedation on mortality in ICU settings. Second, this review included trials from various clinical settings and volatile sedative types, which encompassed different patient populations and enhanced the generalizability of the study findings. Third, we performed multiple sensitivity analyses, all of which confirmed the overall analysis, reinforcing the robustness of the pooled results.

However, this study also has several limitations. First, the included studies exhibited clinical heterogeneity, in terms of disease severity, etiology, and co-intervention, and were published over three decades. Moreover, the most recent and largest RCT of sevoflurane in 687 ARDS patients [18] had a substantial impact on the pooled results. Even though the sensitivity analyses, including a Bayesian hierarchical model, supported the main analysis and no strong evidence for between-study heterogeneity was detected (i.e., I^2^ = 0%, Tau^2^ = 0, posterior standard deviation τ = 0.09), the generalizability of the mortality findings may be limited. The unique features of this specific trial (e.g., exclusive recruitment of patients with moderate to severe ARDS and frequent use of neuromuscular blockade) may interact with the effects of volatile sedation in ways not applicable to other clinical scenarios. Nevertheless, most sensitivity and subgroup analyses that accounted for between-study variations—even those excluding this largest trial—yielded point estimates similar to the primary results, suggesting the robustness of our findings. Second, the included studies assessed mortality at different timepoints, ranging from 24 h to 90 days (Supplementary Table S3). However, pooling mortality data at different timepoints did not alter the point estimates but increased statistical power [57]. Third, only about one third of the included studies were judged as overall low risk-of-bias. However, a sensitivity analysis confined to these high-quality studies yielded results consistent with the primary analysis. Fourth, this review could not evaluate the economic and environmental impacts of volatile sedation, as no data on these outcomes were available. The safe delivery of volatile sedation in the ICU requires specialized equipment, which is associated with increased healthcare costs [58]. In addition, use of volatile anesthetics may contribute to global warming [59]. Given the importance of these factors in assessing the sustainability of volatile sedation in the ICU [60], future research should include these outcomes to inform clinical decision-making. Fifth, given the variations in the type of volatile and IV anesthetics across the include studies, a network meta-analysis might be a suitable option to address our research question. However, substantial clinical and methodological heterogeneity among the studies was expected, making the conduct infeasible.

## Conclusions

This systematic review and meta-analysis suggest that, compared to IV sedation, volatile sedation may increase mortality in mechanically ventilated critically ill adults. The current body of evidence was mainly driven by a recent large trial assessing sevoflurane in patients with moderate to severe ARDS, limiting the generalizability in other settings. Therefore, the use of volatile sedation in the ICU should only be considered in limited clinical scenarios, such as refractory bronchospasm in severe asthma, inadequate sedation despite optimal IV therapy, or limited availability of IV sedatives. Future research should focus on identifying the optimal patient selection and administration regimen to maximize benefits while minimizing harm.

## Supplementary Information

Supplementary meterial 1

## Data Availability

We collected the summary data from published manuscripts. The published article and its supplementary files include all the data generated or analyzed for this study. Further information is available from the corresponding authors upon reasonable request.

## List of abbreviations

ARDS: Acute respiratory distress syndrome
CI: Confidence interval
COVID-19: Coronavirus disease 2019
GRADE: Grading of recommendations assessment, development and evaluation
ICU: Intensive care unit
IV: Intravenous
PONV: Postoperative nausea and vomiting
PRISMA: Preferred reporting items for systematic reviews and meta-analyses
RCT: Randomized controlled trial
RR: Relative risk
SESAR: Sevoflurane for sedation in ARDS
TSA: Trial sequential analysis
