# Supplementary material for: Volatile sedation in critically ill adults undergoing mechanical ventilation: a meta-analysis of randomized controlled trials
Source: Crit Care. 2025 Jun 5;29:227. doi: 10.1186/s13054-025-05467-8 (PMC12142876; doi:10.1186/s13054-025-05467-8)
Supplement: Supplementary file 1 — Supplementary meterial 1 [file 13054_2025_5467_MOESM1_ESM.docx]

Supplemental Material

Volatile sedation in critically ill adults undergoing mechanical ventilation: a meta-analysis of randomized controlled trials

Authors

Taihei Yamamoto, MD, Yuki Kotani, MD, Koya Akutagawa, MD, Tomohisa Nagayama, MD, Maho Tomimatsu, MD, Mayuko Tonai, MD, Toshiyuki Karumai, MD, Yoshiro Hayashi, MD, PhD

Table of contents

[Search strategy for systematic literature review 4](#_Toc196511263)

[PRISMA 2020 checklist 6](#_Toc196511264)

[Supplemental Figure S1. Funnel plot for mortality at the longest follow-up 9](#_Toc196511265)

[Supplemental Figure S2. Forest plot for mortality at the longest follow-up in studies using propofol as comparator. 10](#_Toc196511266)

[Supplemental Figure S3. Forest plot for mortality at the longest follow-up in acute respiratory distress syndrome. 11](#_Toc196511267)

[Supplemental Figure S4. Forest plot for mortality at the longest follow-up in surgical versus non-surgical settings. 12](#_Toc196511268)

[Supplemental Figure S5. Forest plot for mortality at the longest follow-up in overall low risk of bias studies. 13](#_Toc196511269)

[Supplemental Figure S6. Forest plot for mortality at the longest follow-up according to volatile anesthetic delivery device. 14](#_Toc196511270)

[Supplemental Figure S7. Forest plot for mortality at the longest follow-up based on COVID-19 status. 15](#_Toc196511271)

[Supplemental Figure S8. Forest plot for mortality at the longest follow-up based on sedation duration. 16](#_Toc196511272)

[Supplemental Figure S9. Forest plot for duration of mechanical ventilation. 17](#_Toc196511273)

[Supplemental Figure S10. Forest plot for hospital stay. 18](#_Toc196511274)

[Supplemental Figure S11. Forest plot for intensive care unit stay. 19](#_Toc196511275)

[Supplemental Figure S12 Forest plot for hypotension. 20](#_Toc196511276)

[Supplemental Figure S13. Trial sequential analysis of length of intensive care unit stay. 21](#_Toc196511277)

[Supplemental Figure S14. Forest plot for atrial fibrillation. 22](#_Toc196511278)

[Supplemental Figure S15. Forest plot for acute kidney injury. 23](#_Toc196511279)

[Supplemental Figure S16. Forest plot for delirium. 24](#_Toc196511280)

[Supplemental Figure S17. Forest plot for postoperative nausea and vomiting. 25](#_Toc196511281)

[Supplemental Figure S18. Trial sequential analysis of acute kidney injury. 26](#_Toc196511282)

[Supplemental Figure S19. Trial sequential analysis of delirium. 27](#_Toc196511283)

[Supplementary Figure S20. Forest plot for time to extubation from sedation termination. 28](#_Toc196511284)

[Table S1. Major exclusions and reasons for exclusion, in order of year of publication. 29](#_Toc196511285)

[Table S2. Risk of bias assessment of included studies. 31](#_Toc196511286)

[Table S3. Timepoints of mortality assessment. 32](#_Toc196511287)

[Table S4. GRADE evaluation 33](#_Toc196511288)

[Table S5. Summary of sensitivity analyses for secondary outcomes 36](#_Toc196511289)

[Table S5. Summary of sensitivity analyses for secondary outcomes 44](#_Toc196511290)

[Table S6. Summary of additional sensitivity analyses for secondary outcomes 48](#_Toc196511291)

[Changes from the original review protocol 51](#_Toc196511292)

[Supplementary references 52](#_Toc196511293)

# **Search strategy for systematic literature review**

PubMed

(volatile [tiab] OR halogenated [tiab] OR inhal*[tiab] OR sevofl*[tiab] OR isofl*[tiab] OR desfl*[tiab]) AND (randomized controlled trial [pt] OR controlled clinical trial [pt] OR randomized controlled trials[mh] OR random allocation [mh] OR double-blind method [mh] OR single-blind method [mh] OR clinical trial [pt] OR clinical trials [mh] OR clinical trial [tw] OR placebos [mh] OR placebo*[tw] OR random*[tw] OR follow-up studies [mh] OR prospective studies [mh] OR cross-over studies [mh] OR control*[tw] OR prospectiv*[tw] OR randomized [tiab] or randomization [TIAB] or randomly [TIAB] or randomised prospective study [tiab] or post hoc analysis [tiab] or post hoc analyses [tiab] or assigned receive [tiab] or cluster-randomized [tiab]) AND (intensive care unit [tiab] OR critical care [tiab] OR Critically ill [tiab] OR Critically ill patien* [tiab] OR Critical Care [mh] OR Critical Illness [mh] OR Intensive Care Units [mh] OR Postoperative Care [mh] OR cardiogenic shock [tiab] OR targeted temperature management [tiab] or ICU [tiab]) NOT (animal [mh] NOT human [mh] OR comment [pt] OR editorial [pt] OR meta-analysis [pt] OR practice-guideline [pt] OR review [pt] or Models, Animal [mh])

Cochrane Library

#1 "randomized controlled trial":ti,ab,kw or "controlled clinical trial":ti,ab,kw or mh "randomized controlled trials" or mh "random allocation" or mh "double-blind method" or mh "single-blind method" or "clinical trial":ti,ab,kw or mh "clinical trials" or "clinical trial":ti,ab,kw or mh "placebos" or (NEXT placebo*):ti,ab,kw or (NEXT random*):ti,ab,kw or mh "follow-up studies" or mh "prospective studies" or mh "cross-over studies" or (NEXT control*):ti,ab,kw or (NEXT prospectiv*):ti,ab,kw or "randomized":ti,ab,kw or "randomization":ti,ab,kw or "randomly":ti,ab,kw or "randomised prospective study":ti,ab,kw or "post hoc analysis":ti,ab,kw or "post hoc analyses":ti,ab,kw or "assigned receive":ti,ab,kw or "cluster-randomized":ti,ab,kw

#2 "intensive care unit":ti,ab,kw or "critical care":ti,ab,kw or "critically ill":ti,ab,kw or (critically ill NEXT patien*):ti,ab,kw or mh "Critical Care" or mh "Critical Illness" or mh "Intensive Care Units" or mh "Postoperative Care" OR "cardiogenic shock":ti,ab,kw OR "targeted temperature management":ti,ab,kw

#3 "volatile":ti,ab,kw OR "halogenated":ti,ab,kw OR (NEXT inhal*):ti,ab,kw OR (NEXT sevofl*):ti,ab,kw OR (NEXT isofl*):ti,ab,kw OR (NEXT desfl*):ti,ab,kw

#4 mh "Animals" not mh "Humans" or comment:ti,ab,kw or editorial:ti,ab,kw or meta-analysis:ti,ab,kw or practice-guideline:ti,ab,kw or review:ti,ab,kw

#5 #1 and #2 and #3 not #4

Embase

('volatile':ab,ti OR 'halogenated':ab,ti OR 'inhal*':ab,ti OR 'sevofl*':ab,ti OR 'isofl*':ab,ti OR 'desfl*':ab,ti) AND ('randomized controlled trial':it OR 'controlled clinical trial':it OR 'randomized controlled trials':de OR 'random allocation':de OR 'double-blind method':de OR 'single-blind method':de OR 'clinical trial':it OR 'clinical trials':de OR 'clinical trial':ti,ab,kw OR 'placebos':de OR 'placebo*':ti,ab,kw OR 'random*':ti,ab,kw OR 'follow-up studies':de OR 'prospective studies':de OR 'cross-over studies':de OR 'control*':ti,ab,kw OR 'prospectiv*':ti,ab,kw OR 'randomized':ti,ab,kw OR 'randomization':ti,ab,kw OR 'randomly':ti,ab,kw OR 'randomised prospective study':ti,ab,kw OR 'post hoc analysis':ti,ab,kw OR 'post hoc analyses':ti,ab,kw OR 'random number table':ti,ab,kw OR 'assigned receive':ti,ab,kw OR 'cluster-randomized':ti,ab,kw) AND ('intensive care unit':ti,ab OR 'critically ill':ti,ab,kw OR 'critically ill patien*':ti,ab,kw OR 'critical care':ti,ab,kw OR 'critical illness':ti,ab,kw OR 'intensive care units':ti,ab,kw OR 'postoperative care':ti,ab,kw OR 'cardiogenic shock':ab,ti OR 'targeted temperature management':ab,ti) NOT (animal:de NOT human:de OR comment:it OR editorial:it OR 'meta analysis':it OR 'practice guideline':it OR review:it)

# **PRISMA 2020 checklist**

| **Section and Topic** | **Item #** | **Checklist item** | **Location where item is reported** |
| --- | --- | --- | --- |
| **TITLE** | | |  |
| Title | 1 | Identify the report as a systematic review. | 1 |
| **ABSTRACT** | | |  |
| Abstract | 2 | See the PRISMA 2020 for Abstracts checklist. | 2 |
| **INTRODUCTION** | | |  |
| Rationale | 3 | Describe the rationale for the review in the context of existing knowledge. | 4 |
| Objectives | 4 | Provide an explicit statement of the objective(s) or question(s) the review addresses. | 4 |
| **METHODS** | | |  |
| Eligibility criteria | 5 | Specify the inclusion and exclusion criteria for the review and how studies were grouped for the syntheses. | 5 |
| Information sources | 6 | Specify all databases, registers, websites, organisations, reference lists and other sources searched or consulted to identify studies. Specify the date when each source was last searched or consulted. | 5 |
| Search strategy | 7 | Present the full search strategies for all databases, registers and websites, including any filters and limits used. | Supplemental Material |
| Selection process | 8 | Specify the methods used to decide whether a study met the inclusion criteria of the review, including how many reviewers screened each record and each report retrieved, whether they worked independently, and if applicable, details of automation tools used in the process. | 5 |
| Data collection process | 9 | Specify the methods used to collect data from reports, including how many reviewers collected data from each report, whether they worked independently, any processes for obtaining or confirming data from study investigators, and if applicable, details of automation tools used in the process. | 6 |
| Data items | 10a | List and define all outcomes for which data were sought. Specify whether all results that were compatible with each outcome domain in each study were sought (e.g. for all measures, time points, analyses), and if not, the methods used to decide which results to collect. | 6 |
|  | 10b | List and define all other variables for which data were sought (e.g. participant and intervention characteristics, funding sources). Describe any assumptions made about any missing or unclear information. | 6 |
| Study risk of bias assessment | 11 | Specify the methods used to assess risk of bias in the included studies, including details of the tool(s) used, how many reviewers assessed each study and whether they worked independently, and if applicable, details of automation tools used in the process. | 6 |
| Effect measures | 12 | Specify for each outcome the effect measure(s) (e.g. risk ratio, mean difference) used in the synthesis or presentation of results. | 6-7 |
| Synthesis methods | 13a | Describe the processes used to decide which studies were eligible for each synthesis (e.g. tabulating the study intervention characteristics and comparing against the planned groups for each synthesis (item #5)). | 6 |
|  | 13b | Describe any methods required to prepare the data for presentation or synthesis, such as handling of missing summary statistics, or data conversions. | 6 |
|  | 13c | Describe any methods used to tabulate or visually display results of individual studies and syntheses. | 6 |
|  | 13d | Describe any methods used to synthesize results and provide a rationale for the choice(s). If meta-analysis was performed, describe the model(s), method(s) to identify the presence and extent of statistical heterogeneity, and software package(s) used. | 6-7 |
|  | 13e | Describe any methods used to explore possible causes of heterogeneity among study results (e.g. subgroup analysis, meta-regression). | 7 |
|  | 13f | Describe any sensitivity analyses conducted to assess robustness of the synthesized results. | 7 |
| Reporting bias assessment | 14 | Describe any methods used to assess risk of bias due to missing results in a synthesis (arising from reporting biases). | 6 |
| Certainty assessment | 15 | Describe any methods used to assess certainty (or confidence) in the body of evidence for an outcome. | 6 |
| **RESULTS** | | |  |
| Study selection | 16a | Describe the results of the search and selection process, from the number of records identified in the search to the number of studies included in the review, ideally using a flow diagram. | 7, Fig. 1 |
|  | 16b | Cite studies that might appear to meet the inclusion criteria, but which were excluded, and explain why they were excluded. | 7 |
| Study characteristics | 17 | Cite each included study and present its characteristics. | 7-8 |
| Risk of bias in studies | 18 | Present assessments of risk of bias for each included study. | 8, Supplemental Material |
| Results of individual studies | 19 | For all outcomes, present, for each study: (a) summary statistics for each group (where appropriate) and (b) an effect estimate and its precision (e.g. confidence/credible interval), ideally using structured tables or plots. | 7-8, Table 1 |
| Results of syntheses | 20a | For each synthesis, briefly summarise the characteristics and risk of bias among contributing studies. | 7, Table 1, Supplemental Material |
|  | 20b | Present results of all statistical syntheses conducted. If meta-analysis was done, present for each the summary estimate and its precision (e.g. confidence/credible interval) and measures of statistical heterogeneity. If comparing groups, describe the direction of the effect. | 8-9, Table 2, Table 3 |
|  | 20c | Present results of all investigations of possible causes of heterogeneity among study results. | 8-9, Table 2, Table 3 |
|  | 20d | Present results of all sensitivity analyses conducted to assess the robustness of the synthesized results. | 8-9, Table 2, Supplemental Material |
| Reporting biases | 21 | Present assessments of risk of bias due to missing results (arising from reporting biases) for each synthesis assessed. | Supplemental Material |
| Certainty of evidence | 22 | Present assessments of certainty (or confidence) in the body of evidence for each outcome assessed. | Supplemental Material |
| **DISCUSSION** | | |  |
| Discussion | 23a | Provide a general interpretation of the results in the context of other evidence. | 9 |
|  | 23b | Discuss any limitations of the evidence included in the review. | 12-13 |
|  | 23c | Discuss any limitations of the review processes used. | 12-13 |
|  | 23d | Discuss implications of the results for practice, policy, and future research. | 11-12 |
| **OTHER INFORMATION** | | |  |
| Registration and protocol | 24a | Provide registration information for the review, including register name and registration number, or state that the review was not registered. | 5 |
|  | 24b | Indicate where the review protocol can be accessed, or state that a protocol was not prepared. | 5 |
|  | 24c | Describe and explain any amendments to information provided at registration or in the protocol. | Supplemental Material |
| Support | 25 | Describe sources of financial or non-financial support for the review, and the role of the funders or sponsors in the review. | 14 |
| Competing interests | 26 | Declare any competing interests of review authors. | 14 |
| Availability of data, code and other materials | 27 | Report which of the following are publicly available and where they can be found: template data collection forms; data extracted from included studies; data used for all analyses; analytic code; any other materials used in the review. | 14 |

# **Supplemental Figure S1. Funnel plot for mortality at the longest follow-up**


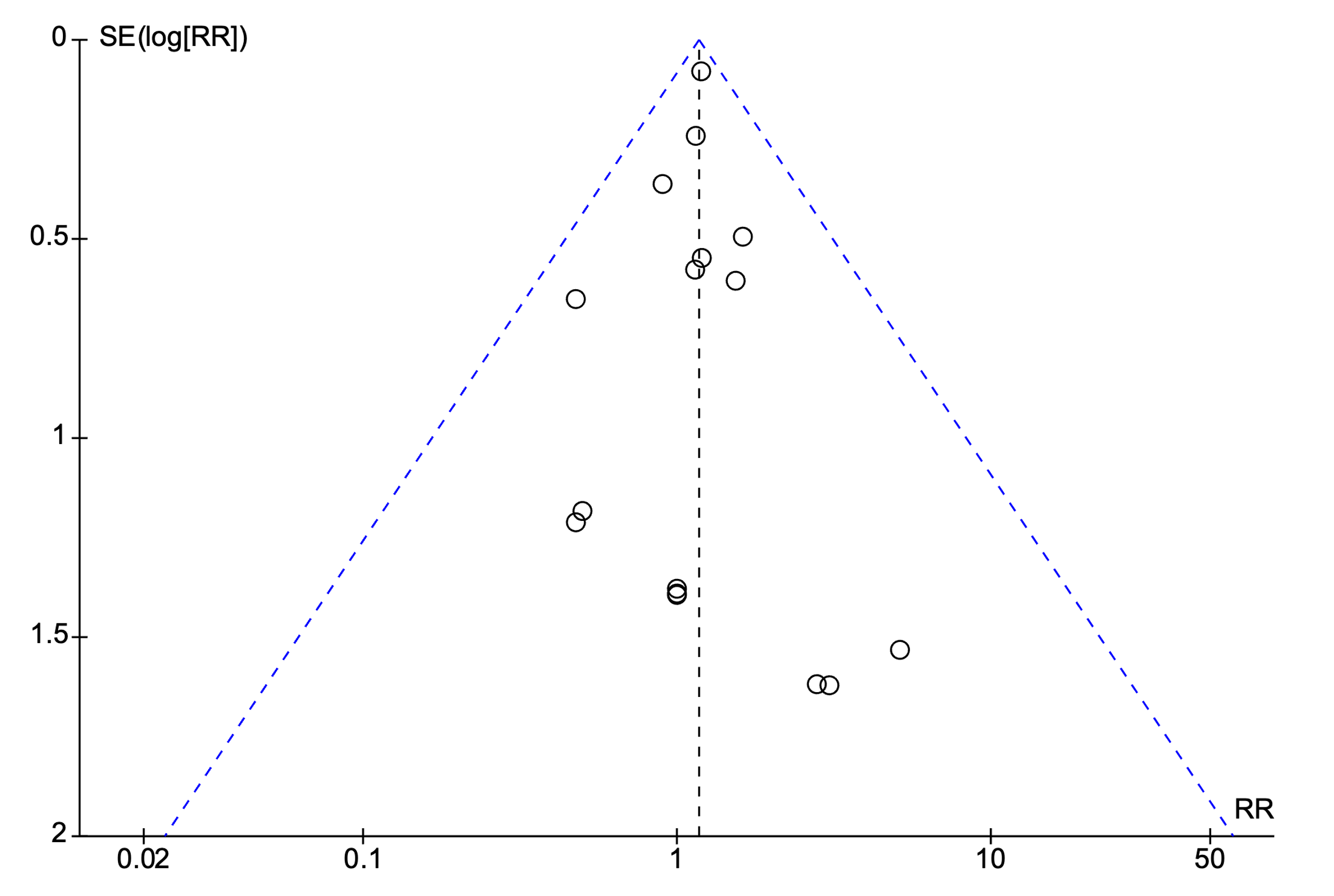


Egger’s test showed no significant evidence of small-study effects (bias coefficient = –0.064 ± 0.186; P = 0.73).

# **Supplemental Figure S2. Forest plot for mortality at the longest follow-up in studies using propofol as comparator.**

**
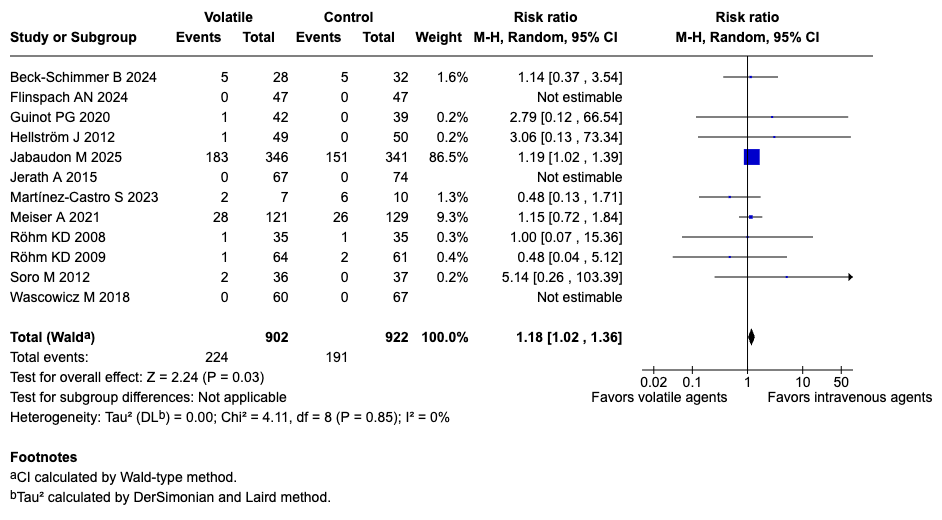
**

# **Supplemental Figure S3. Forest plot for mortality at the longest follow-up in acute respiratory distress syndrome.**

**
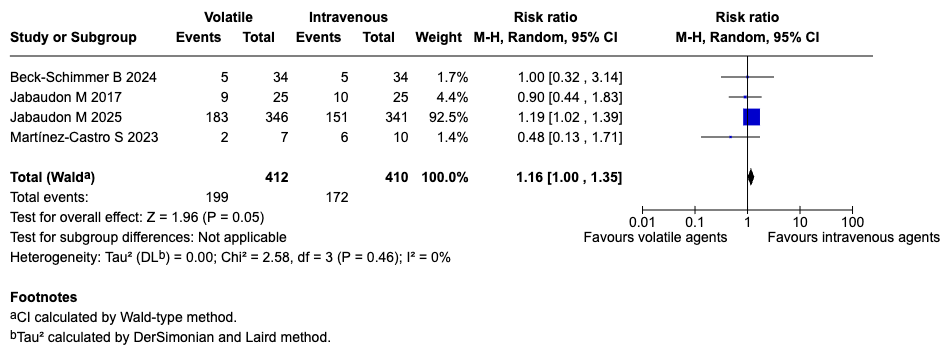
**

# **Supplemental Figure S4. Forest plot for mortality at the longest follow-up in surgical versus non-surgical settings.**

**
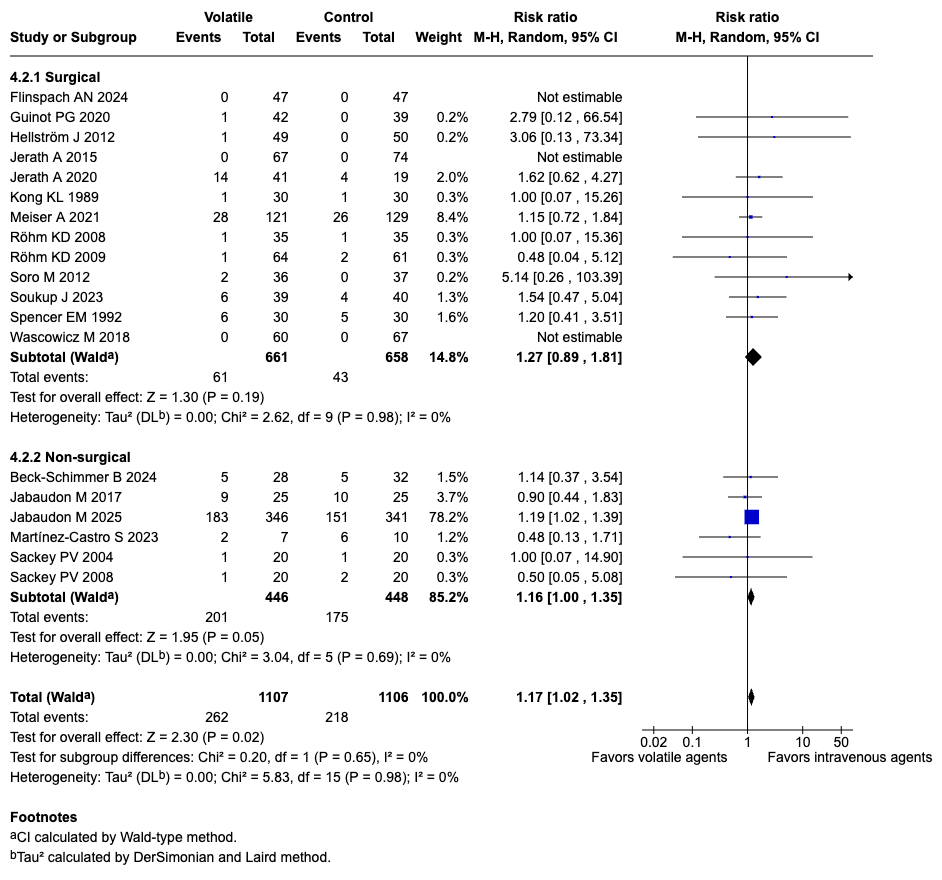
**

# **Supplemental Figure S5. Forest plot for mortality at the longest follow-up in overall low risk of bias studies.**

**
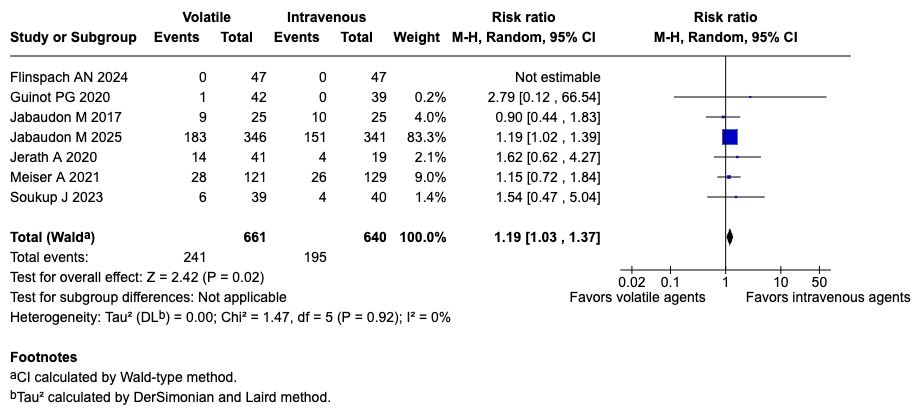
**

# **Supplemental Figure S6. Forest plot for mortality at the longest follow-up according to volatile anesthetic delivery device.**


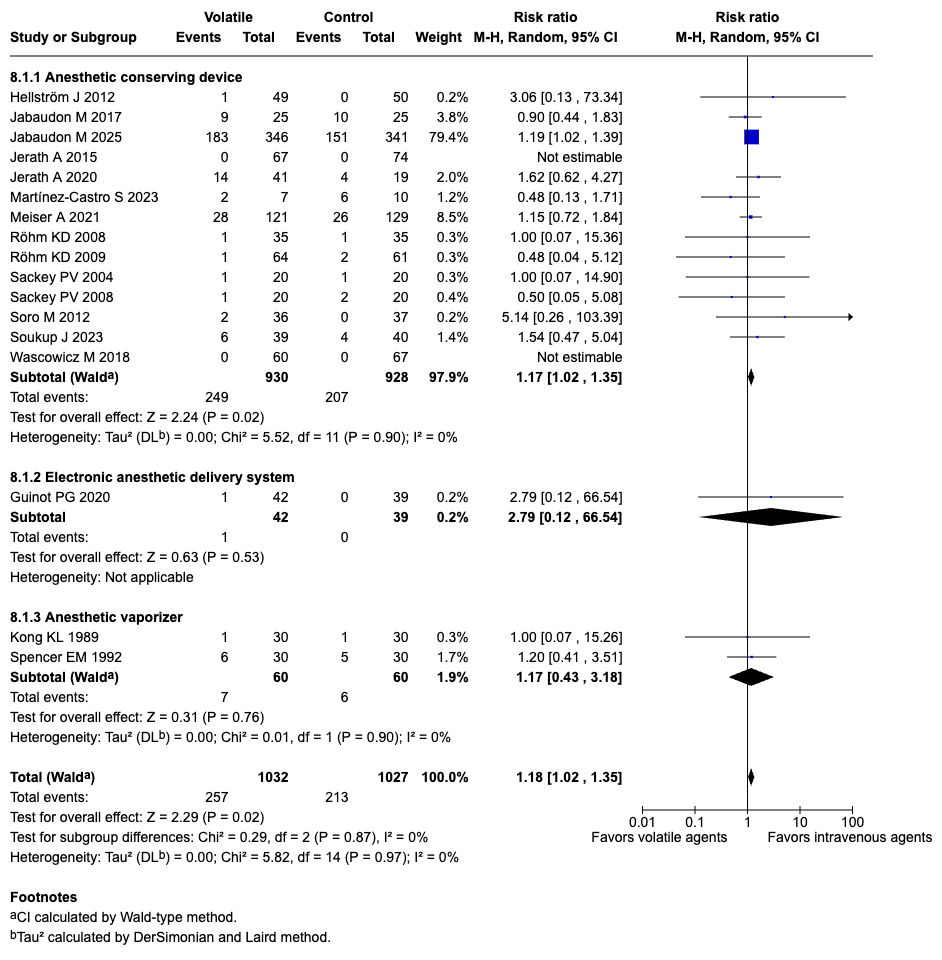


# **Supplemental Figure S7. Forest plot for mortality at the longest follow-up based on COVID-19 status.**


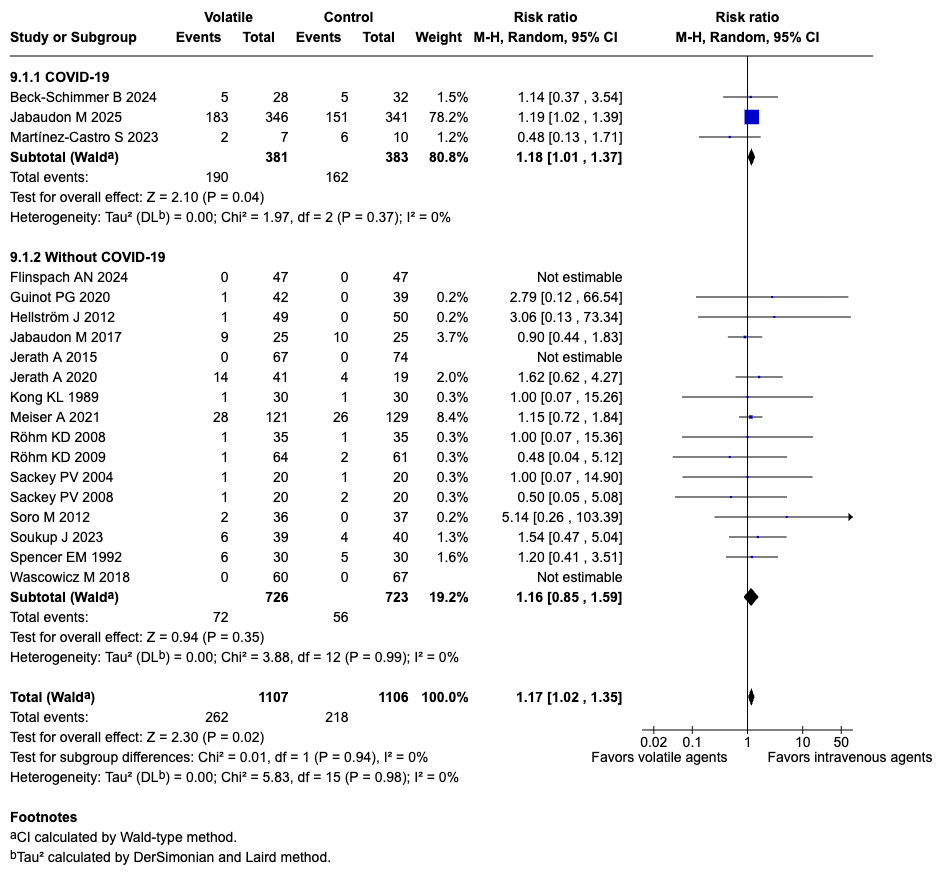


# **Supplemental Figure S8. Forest plot for mortality at the longest follow-up based on sedation duration.**

**
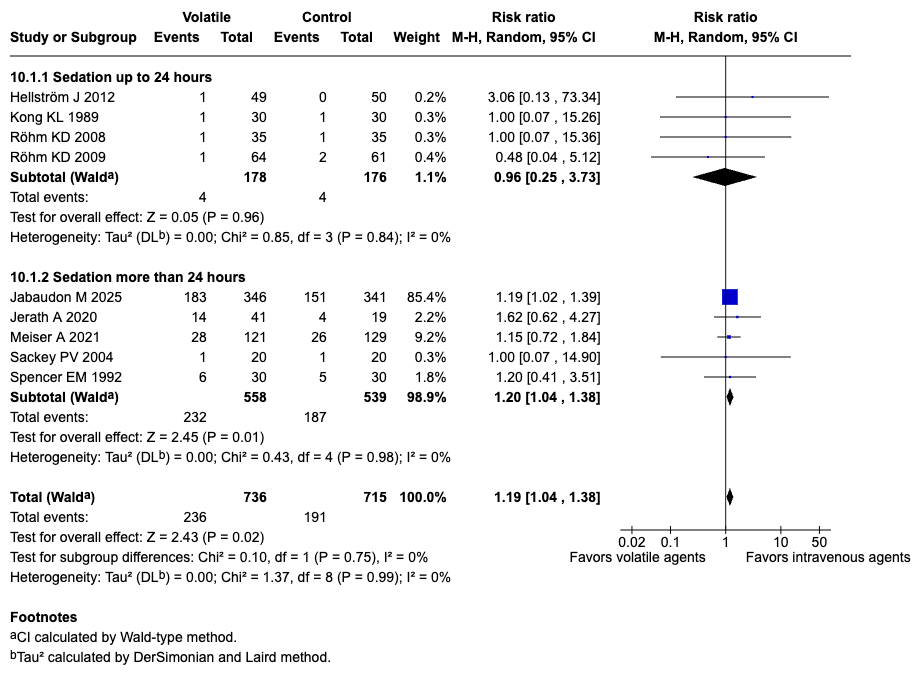
**

# **Supplemental Figure S9. Forest plot for duration of mechanical ventilation.**

**
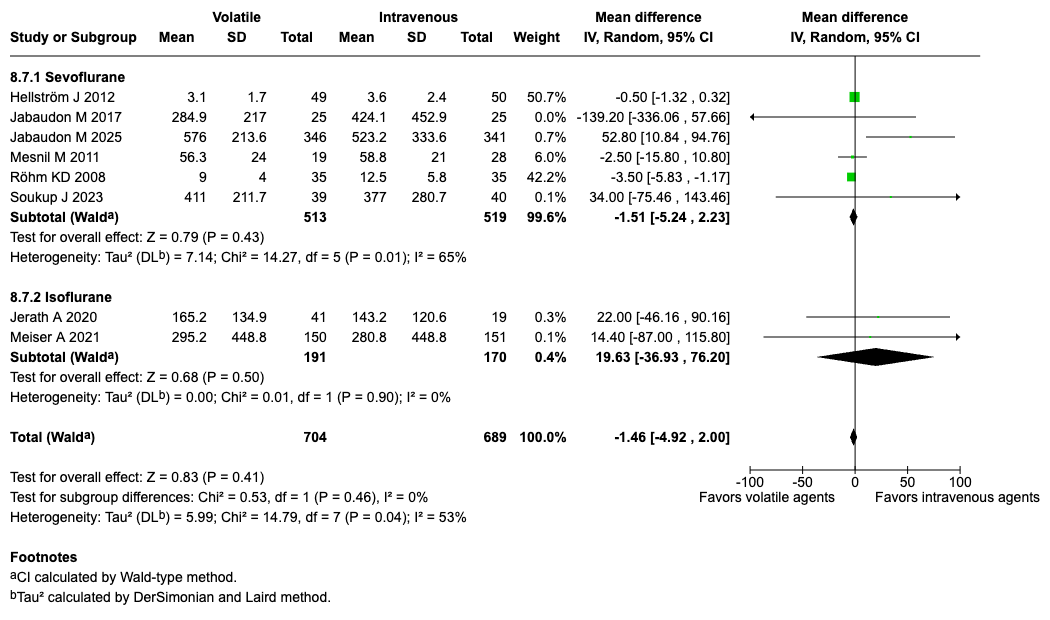
**

# **Supplemental Figure S10. Forest plot for hospital stay.**


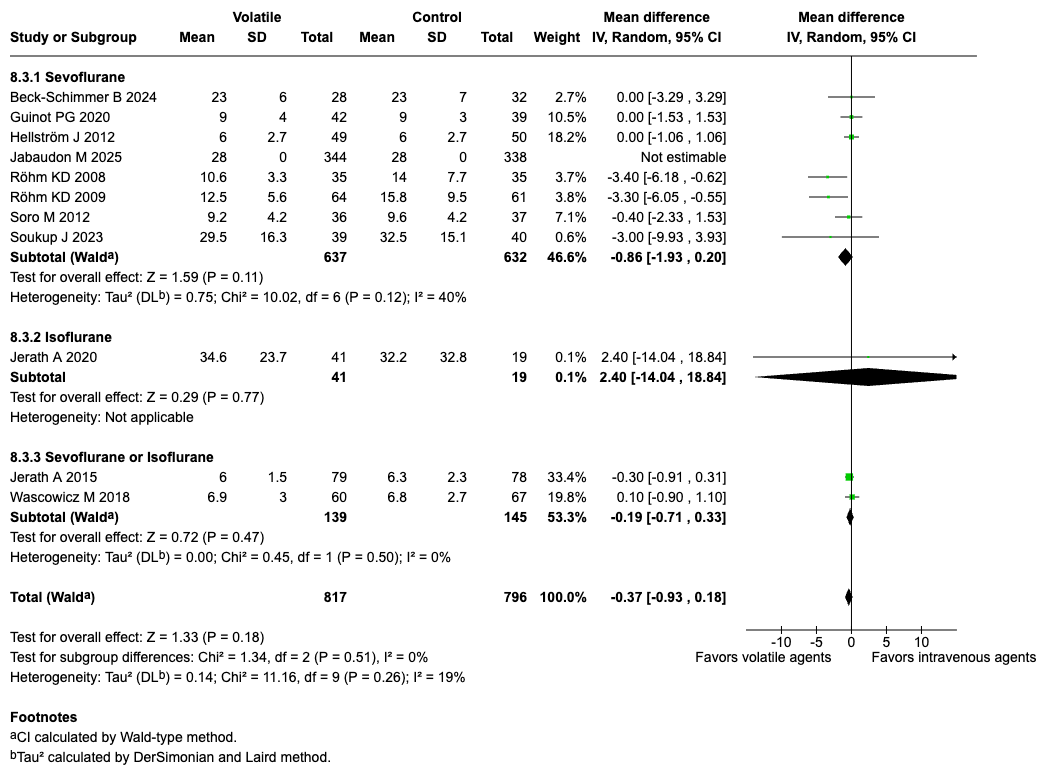


# **Supplemental Figure S11. Forest plot for intensive care unit stay.**

**
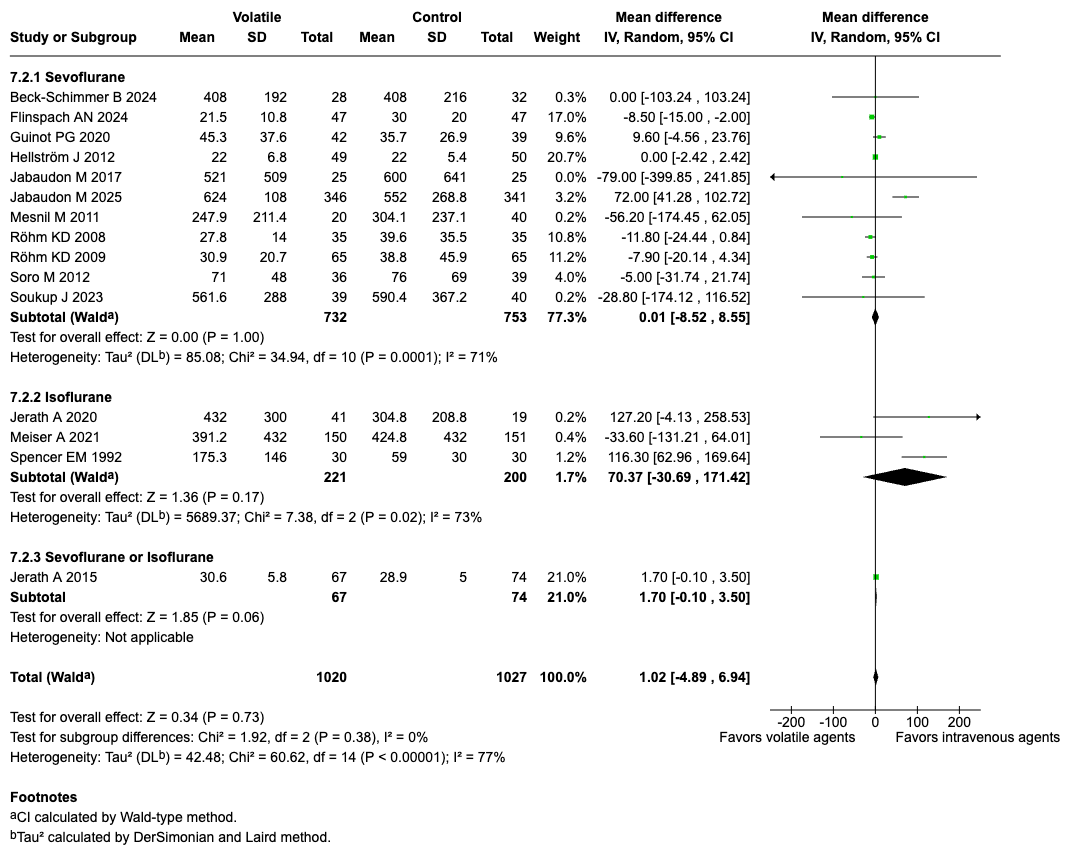
**

# **Supplemental Figure S12 Forest plot for hypotension.**

**
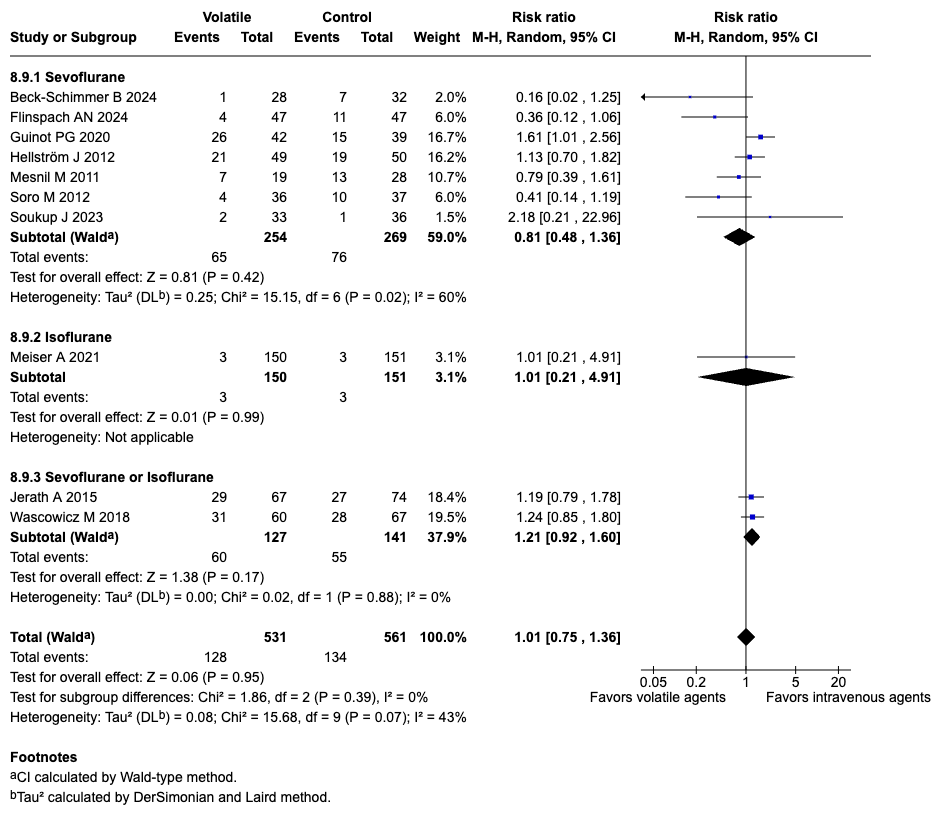
**

# **Supplemental Figure S13. Trial sequential analysis of length of intensive care unit stay.**


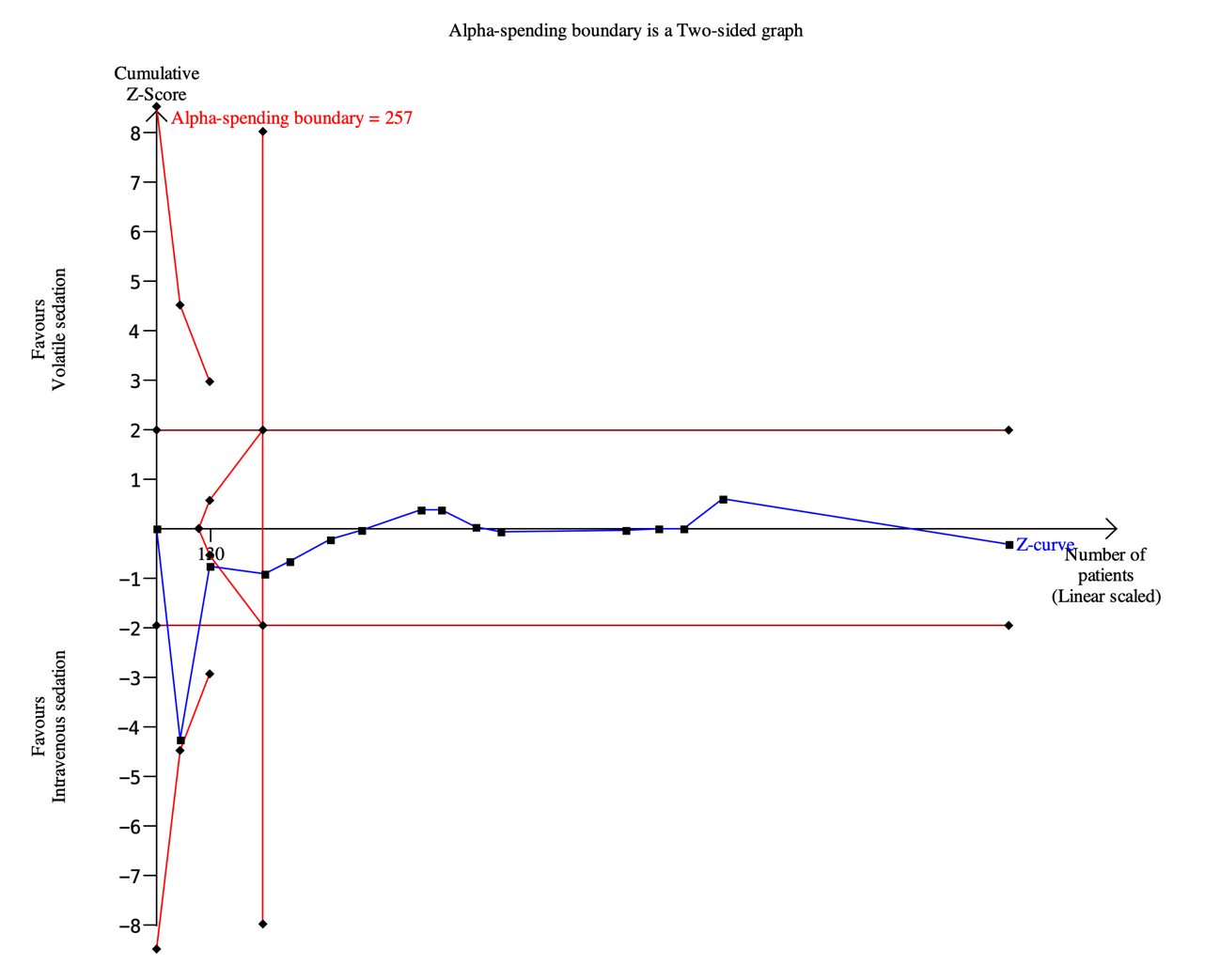


Alpha error = 5%, power = 80%, relative risk decrease = 24 hours, diversity = 0%

# **Supplemental Figure S14. Forest plot for atrial fibrillation.**

**
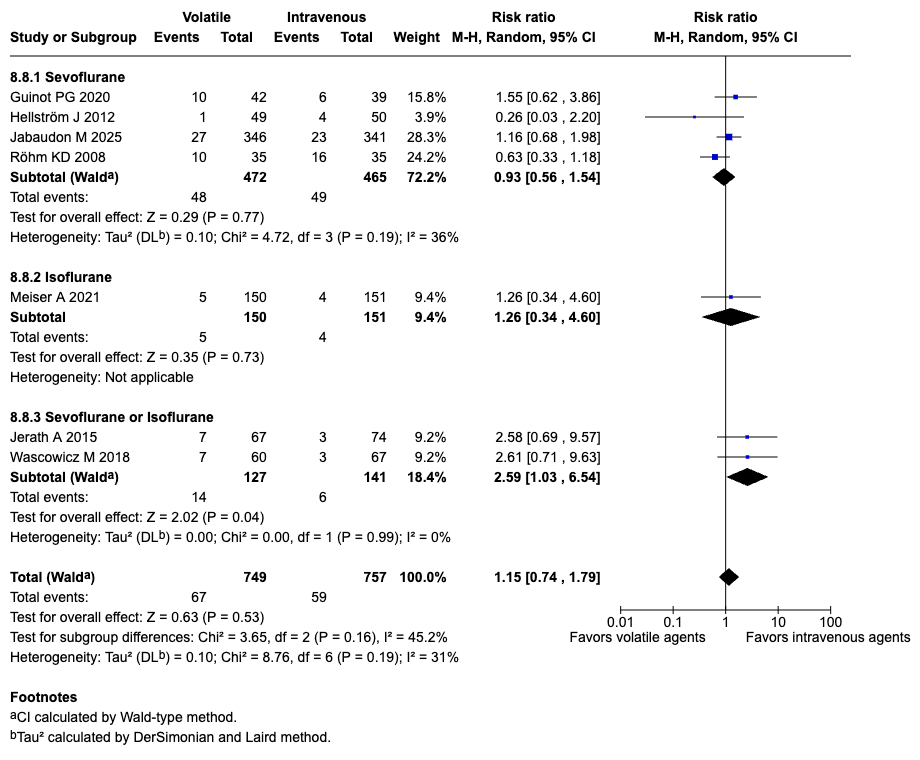
**

# **Supplemental Figure S15. Forest plot for acute kidney injury.**


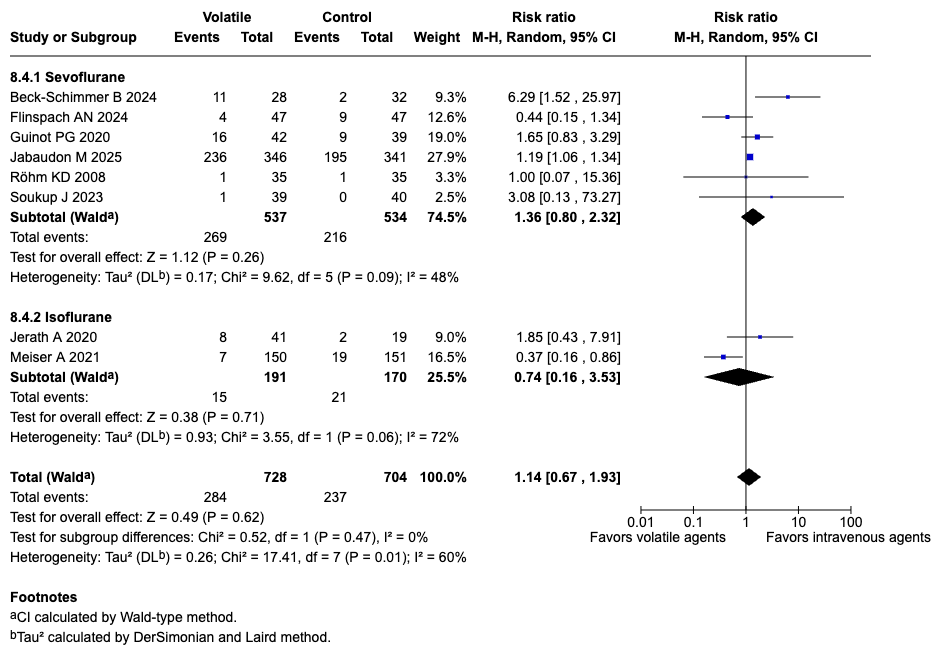


# **Supplemental Figure S16. Forest plot for delirium.**


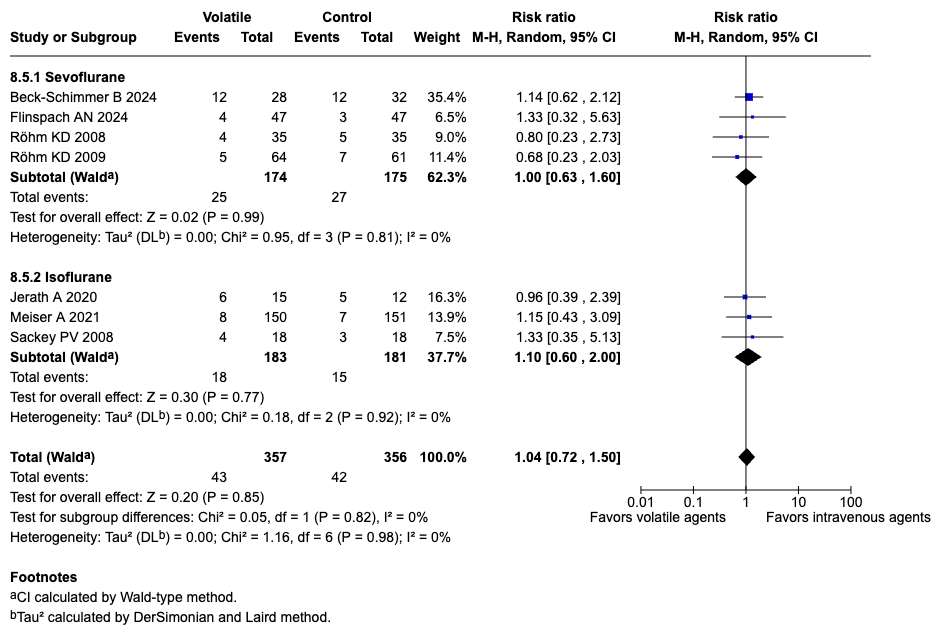


# **Supplemental Figure S17. Forest plot for postoperative nausea and vomiting.**

**
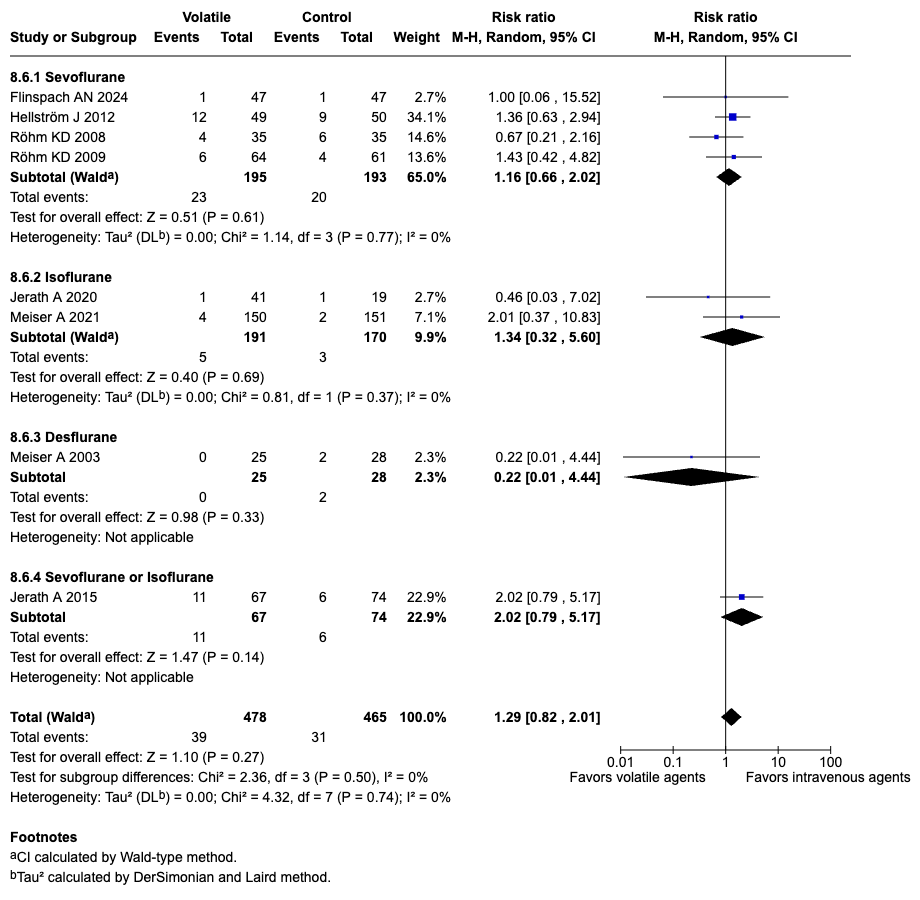
**

# **Supplemental Figure S18. Trial sequential analysis of acute kidney injury.**


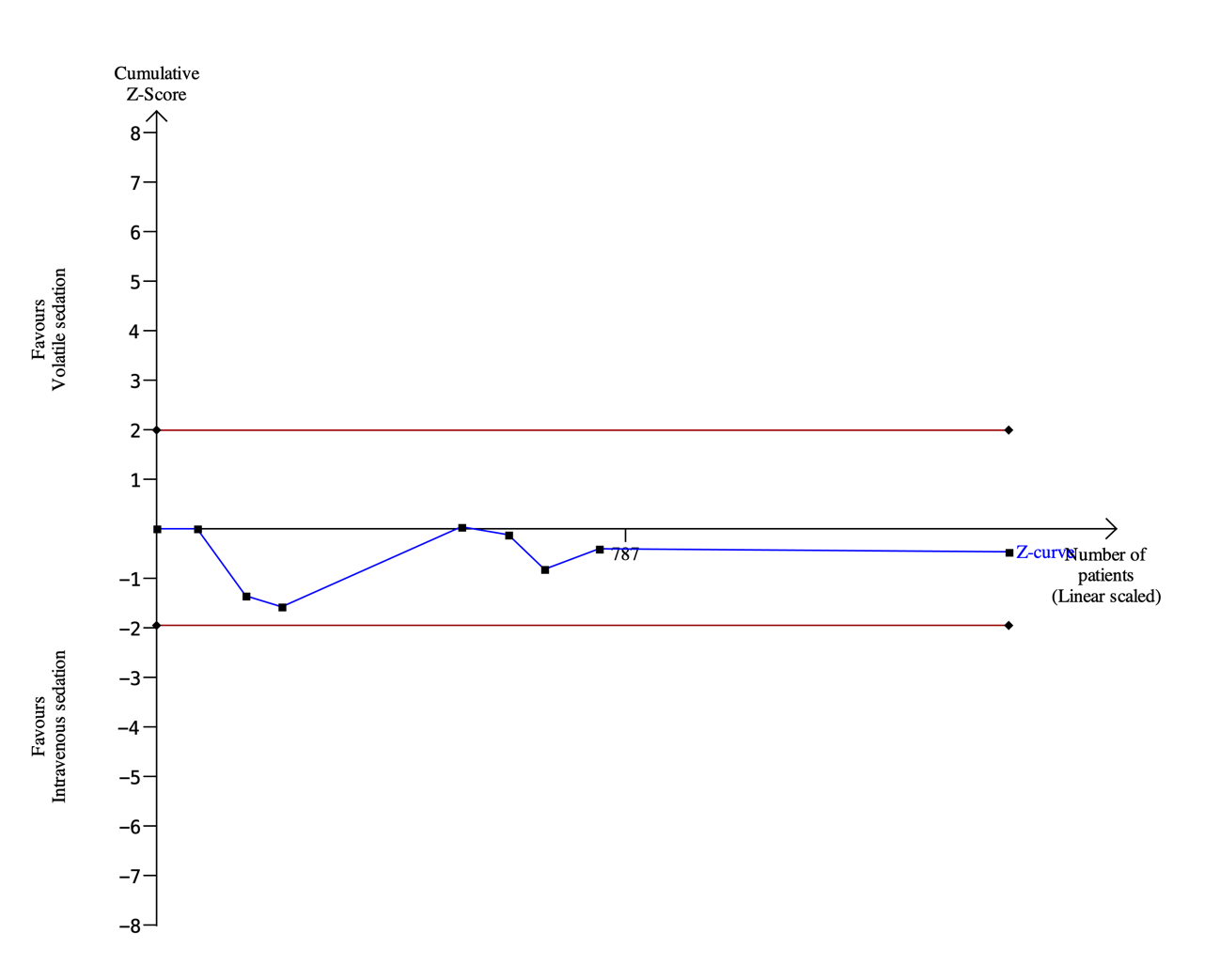


Alpha error = 5%, power = 80%, relative risk decrease = 20%, diversity = 0%

The required information size was 32,010, but it was not shown on the plot due to software settings, as the actual sample size was below the display threshold.

# **Supplemental Figure S19. Trial sequential analysis of delirium.**


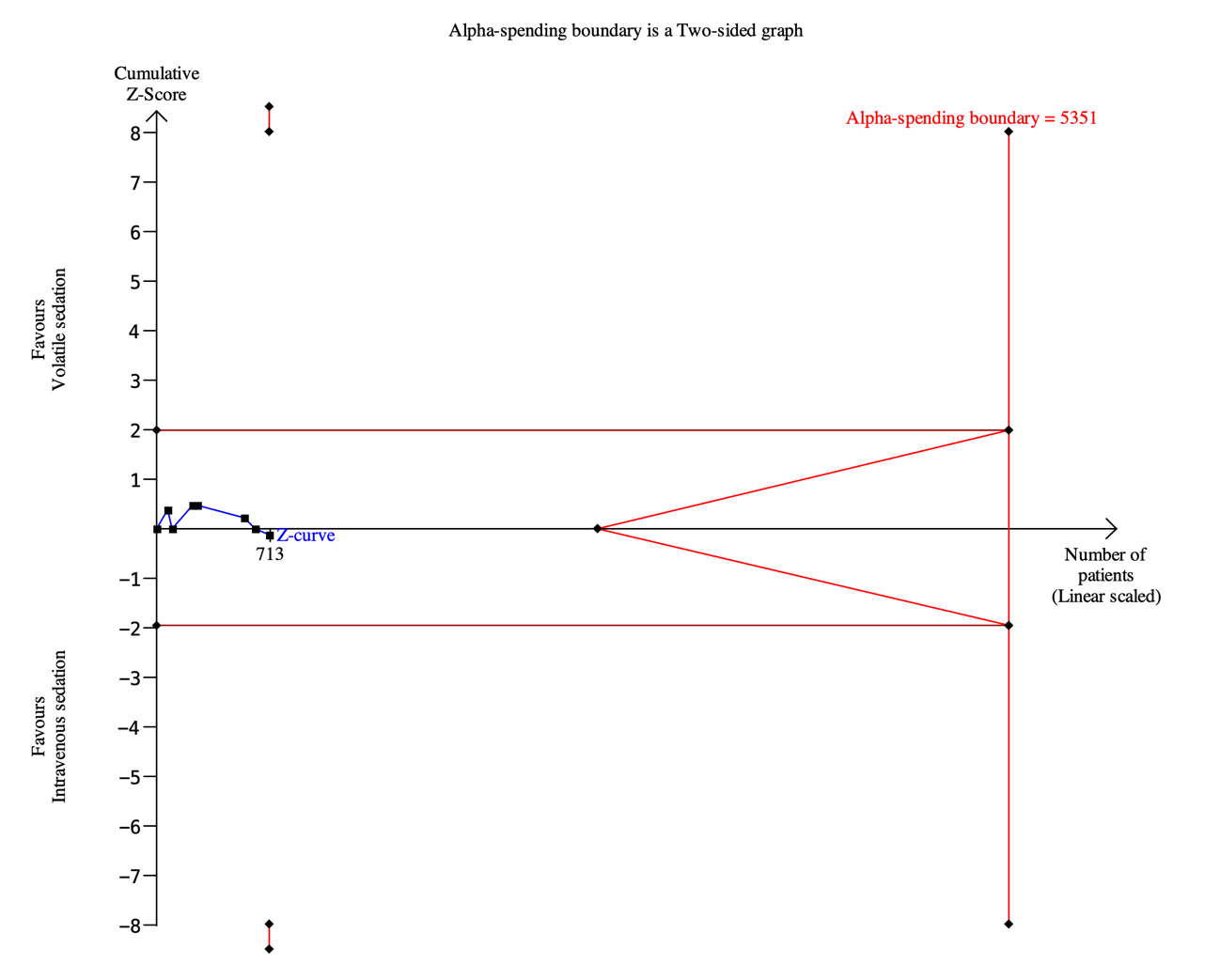


Alpha error = 5%, power = 80%, relative risk decrease = 20%, diversity = 0%

# **Supplementary Figure S20. Forest plot for time to extubation from sedation termination.**


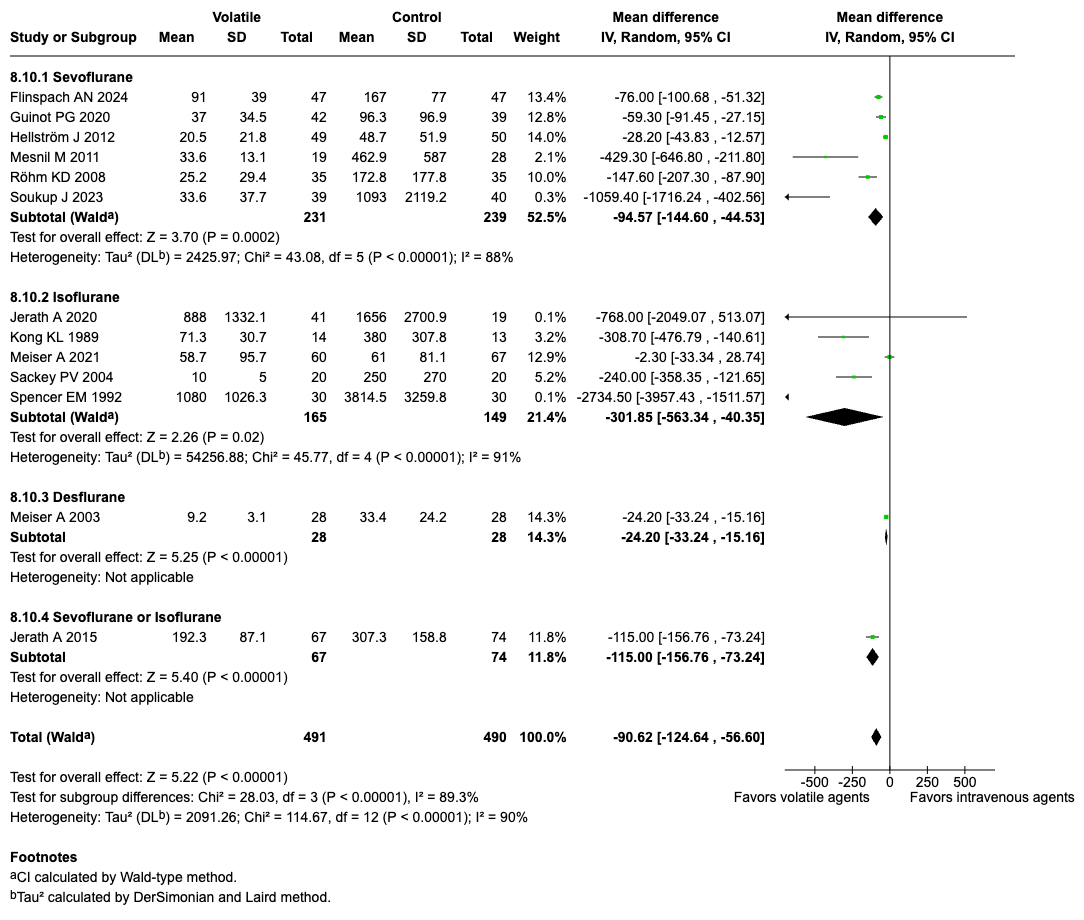


# **Table S1. Major exclusions and reasons for exclusion, in order of year of publication.**

| Author, year | Reason for exclusion |
| --- | --- |
| Spencer EM, 1991[1] | Lack of data on main outcomes |
| Millane TA, 1992[2] | Lack of data on main outcomes |
| Gómez JI, 1995[3] | Unavailable full-text article |
| Tanigami H, 1997[4] | Wrong study design |
| Freye E, 1998[5] | Wrong population |
| Hellström J, 2011[6] | Secondary analysis of an included study |
| Villa F, 2012[7] | Wrong population |
| Steurer MP, 2012[8] | Lack of data on main outcomes |
| Soukup J, 2012[9] | Study protocol |
| Bösel J, 2012[10] | Wrong study design |
| Orriach JL, 2013[11] | Lack of data on main outcomes |
| Wong K, 2014[12] | Congress abstract |
| Ramirez M, 2014[13] | Congress abstract |
| Jerath, A, 2015[14] | Study protocol |
| Bonvini JM, 2015[15] | Secondary analysis of an included study |
| Bellgardt M, 2016[16] | Wrong study design |
| Hassan Wmnw, 2017[17] | Wrong population |
| Staudacher, DL, 2018[18] | Lack of data on main outcomes |
| Meiser A, 2018[19] | Wrong study design |
| Walczak KD, 2019[20] | Secondary analysis of an included study |
| Türktan M, 2019[21] | Lack of data on main outcomes |
| Donadello K, 2019[22] | Congress abstract |
| Sung TY, 2020[23] | Wrong population |
| Jung S, 2020[24] | Wrong study design |
| Bailly P, 2021[25] | Clinical trial registration without data |
| Müller-Wirtz LM, 2022[26] | Secondary analysis of an included study |
| Jabaudon M, 2022[27] | Wrong study design |
| Flinspach AN, 2022[28] | Study protocol |
| Becher T, 2022[29] | Secondary analysis of an included study |
| Bracht H, 2023[30] | Secondary analysis of an included study |
| NCT04415060[31] | Clinical trial registration without data |
| NCT04998253[32] | Clinical trial registration without data |
| NCT05707884[33] | Clinical trial registration without data |

# **Table S2. Risk of bias assessment of included studies.**

**
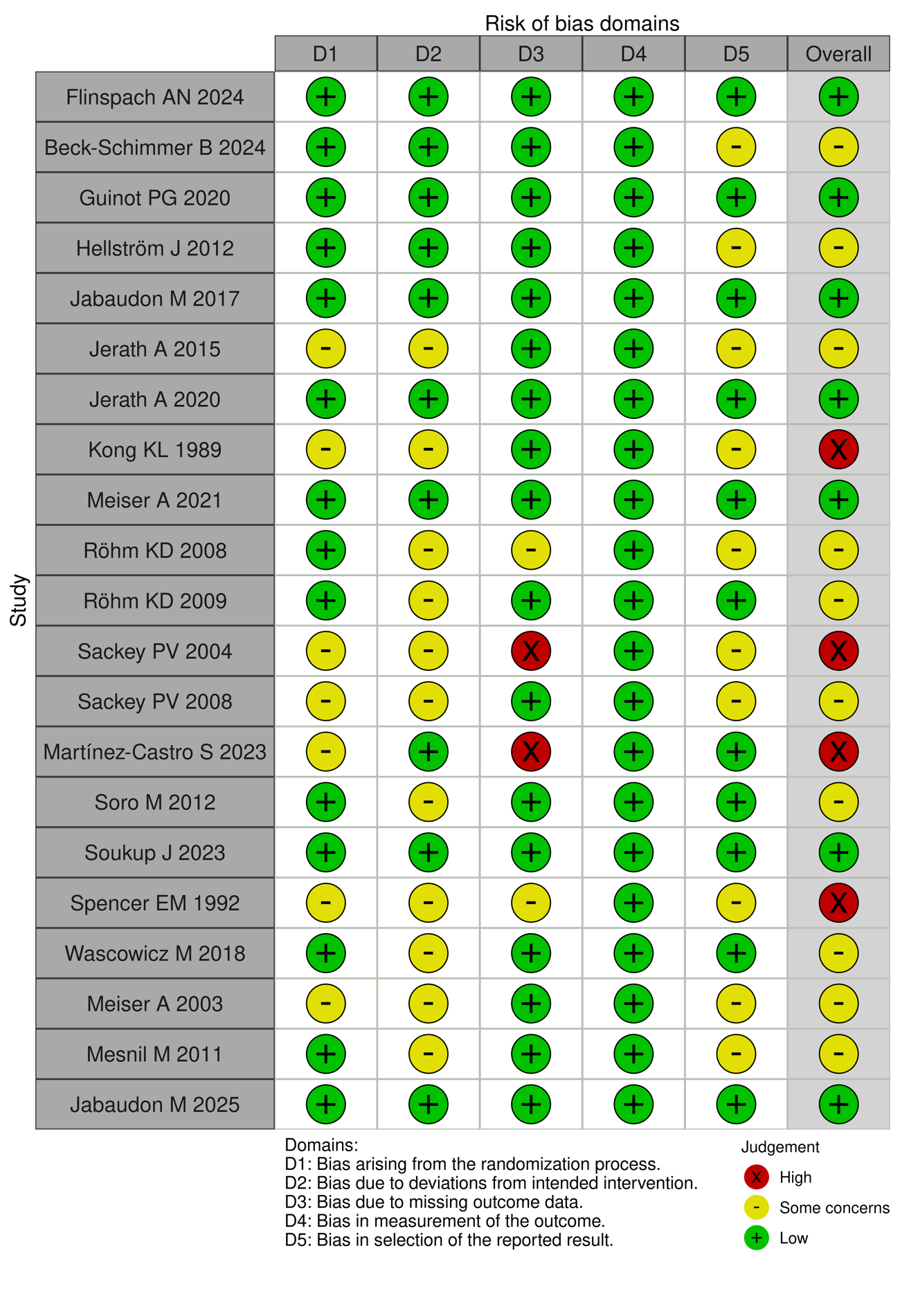
**

# **Table S3. Timepoints of mortality assessment.**

| Timeframe | Number of studies |
| --- | --- |
| 24 hours | 1[34] |
| 4 days | 2[35,36] |
| 7days | 1[1] |
| 28 days | 1[37] |
| 30 days | 5[38–42] |
| 90 days | 1[43] |
| ICU discharge | 1[44] |
| Hospital discharge | 7[14,45–50] |

# **Table S4. GRADE evaluation**

| **Certainty assessment** | | | | | | | **№ of patients** | | **Effect** | | **Certainty** | **Importance** |
| --- | --- | --- | --- | --- | --- | --- | --- | --- | --- | --- | --- | --- |
| **№ of studies** | **Study design** | **Risk of bias** | **Inconsistency** | **Indirectness** | **Imprecision** | **Other considerations** | **Volatile sedation** | **Intravenous sedation** | **Relative (95% CI)** | **Absolute (95% CI)** |  |  |
| **Mortality at the longest follow-up** | | | | | | | | | | | | |
| 19 | randomised trials | serious^a^ | not serious | serious^b^ | not serious | none | 262/1107 (23.7%) | 218/1106 (19.7%) | **RR 1.17** (1.02 to 1.35) | **34 more per 1,000** (from 4 more to 69 more) | ⨁⨁◯◯ Low^a,b^ | CRITICAL |
| **Duration of mechanical ventilation** | | | | | | | | | | | | |
| 8 | randomised trials | not serious | serious^c^ | not serious | not serious | none | 704 | 689 | - | MD **1.46 hour lower** (4.92 lower to 2 higher) | ⨁⨁⨁◯ Moderate^c^ | IMPORTANT |
| **Length of hospital stay** | | | | | | | | | | | | |
| 11 | randomised trials | serious^a^ | not serious | not serious | not serious | none | 817 | 796 | - | MD **0.37 day lower** (0.93 lower to 0.18 higher) | ⨁⨁⨁◯ Moderate^a^ | IMPORTANT |
| **Length of ICU stay** | | | | | | | | | | | | |
| 15 | randomised trials | serious^a^ | serious^d^ | not serious | not serious | none | 1020 | 1027 | - | MD **1.01 hour higher** (4.89 lower to 6.92 higher) | ⨁⨁◯◯ Low^a,d^ | IMPORTANT |
| **Hypotension** | | | | | | | | | | | | |
| 10 | randomised trials | serious^a^ | serious^e^ | not serious | not serious | none | 251/570 (44.0%) | 257/591 (43.5%) | **RR 1.03** (0.84 to 1.26) | **13 more per 1,000** (from 70 fewer to 113 more) | ⨁⨁◯◯ Low^a,e^ | IMPORTANT |
| **Atrial fibrillation** | | | | | | | | | | | | |
| 7 | randomised trials | serious^a^ | serious^d^ | not serious | serious^f^ | none | 67/749 (8.9%) | 59/757 (7.8%) | **RR 1.15** (0.74 to 1.79) | **12 more per 1,000** (from 20 fewer to 62 more) | ⨁◯◯◯ Very low^a,d,f^ | IMPORTANT |
| **Acute kidney injury** | | | | | | | | | | | | |
| 8 | randomised trials | not serious | serious^d,g^ | serious^g^ | serious^f^ | none | 284/728 (39.0%) | 237/704 (33.7%) | **RR 1.14** (0.67 to 1.93) | **47 more per 1,000** (from 111 fewer to 313 more) | ⨁◯◯◯ Very low^d,f,g^ | IMPORTANT |
| **Delirium** | | | | | | | | | | | | |
| 7 | randomised trials | serious^a^ | serious^h^ | not serious | serious^f^ | none | 43/357 (12.0%) | 42/356 (11.8%) | **RR 1.04** (0.72 to 1.50) | **5 more per 1,000** (from 33 fewer to 59 more) | ⨁◯◯◯ Very low^a,f,h^ | IMPORTANT |
| **Postoperative nausea and vomiting** | | | | | | | | | | | | |
| 8 | randomised trials | serious^a^ | serious^i^ | not serious | serious^f^ | none | 39/478 (8.2%) | 31/465 (6.7%) | **RR 1.29** (0.82 to 2.01) | **19 more per 1,000** (from 12 fewer to 67 more) | ⨁◯◯◯ Very low^a,f,i^ | IMPORTANT |
| **Time to extubation from termination of sedation** | | | | | | | | | | | | |
| 13 | randomised trials | serious^a^ | serious^d^ | not serious | not serious | none | 491 | 490 | - | MD **89.13 minutes lower** (125.38 lower to 52.89 lower) | ⨁⨁◯◯ Low^a,d^ | IMPORTANT |

**CI:** confidence interval; **MD:** mean difference; **RR:** risk ratio

#### Explanations

a. Randomization was not properly conducted.

b. Different timepoints were assessed across studies.

c. Criteria to liberation from mechanical ventilation were not standardized across studies.

d. Considerable heterogeneity

e. Diagnosis of hypotension was not standardized across studies.

f. The confidence interval was wide.

g. Diagnosis of acute kidney injury was not standardized across studies.

h. Diagnosis of delirium was not standardized across studies.

i. Diagnosis of postoperative nausea and vomiting was not standardized across studies.

# **Table S5. Summary of sensitivity analyses for secondary outcomes**

| **Secondary outcome** | **No. of studies** | **Volatile sedation** | **Intravenous sedation** | **Relative risk** | **Mean difference** | **95% CI** | **P value** | **I^2^** |
| --- | --- | --- | --- | --- | --- | --- | --- | --- |
| **Duration of mechanical ventilation, hour** | 8 |  |  |  | -1.46 | -4.92 to 2.00 | 0.41 | 53% |
| Sevoflurane | 6 |  |  |  | -1.51 | -5.24 to 2.23 | 0.43 | 65% |
| Isoflurane | 2 |  |  |  | 19.63 | -36.93 to 76.20 | 0.50 | 0% |
| Propofol as comparator | 6 |  |  |  | -1.49 | 1.02 to 1.36 | 0.03 | 0% |
| Surgical settings | 5 |  |  |  | -1.46 | -3.89 to 0.65 | 0.16 | 39% |
| Non-surgical settings | 3 |  |  |  | 10.87 | -44.07 to 65.80 | 0.70 | 75% |
| ARDS | 2 |  |  |  | -18.12 | -199.74 to 163.50 | 0.84 | 71% |
| Anesthetic conserving device  (dead space 100mL) | 7 |  |  |  | -1.65 | -3.85 to 0.55 | 0.14 | 30% |
| Anesthetic conserving device  (dead space 50mL) | 1 |  |  |  | 52.80 | 10.84 to 94.76 | 0.01 | Not applicable |
| COVID-19 | 1 |  |  |  | 52.80 | 10.84 to 94.76 | 0.01 | Not applicable |
| Without COVID-19 | 7 |  |  |  | -1.65 | -3.85 to 0.55 | 0.14 | 30% |
| Sedation up to 24 hours | 2 |  |  |  | -1.79 | -4.70 to 1.12 | 0.23 | 82% |
| Sedation more than 24 hours | 4 |  |  |  | 18.43 | -14.68 to 51.54 | 0.40 | 53% |
| Low risk of bias studies | 5 |  |  |  | 35.74 | 3.96 to 67.52 | 0.03 | 0% |
| **Length of hospital stay, day** | 11 |  |  |  | -0.37 | -0.93 to 0.18 | 0.18 | 19% |
| Sevoflurane | 8 |  |  |  | -0.86 | -1.93 to 0.20 | 0.11 | 40% |
| Isoflurane | 1 |  |  |  | 19.63 | -36.93 to 76.20 | 0.50 | Not applicable |
| Either sevoflurane or isoflurane | 2 |  |  |  | 0.19 | -0.71 to 0.33 | 0.47 | 0% |
| Propofol as comparator | 10 |  |  |  | -0.40 | -0.99 to 0.19 | 0.18 | 28% |
| Surgical settings | 9 |  |  |  | -0.42 | -1.02 to 0.19 | 0.18 | 28% |
| Non-surgical settings | 2 |  |  |  | 0.00 | -3.29 to 3.29 | 1.00 | Not applicable |
| ARDS | 2 |  |  |  | 0.00 | -3.29 to 3.29 | 1.00 | Not applicable |
| Anesthetic conserving device  (dead space 100mL) | 8 |  |  |  | -0.52 | -1.23 to 0.19 | 0.15 | 36% |
| Anesthetic conserving device  (dead space 50mL) | 1 |  |  |  | Not  estimable | Not  estimable | Not  applicable | Not applicable |
| Electronic anesthetic delivery system | 1 |  |  |  | 0.00 | -1.53 to 1.53 | 1.00 | Not applicable |
| Either anesthetic conserving device (dead space 100mL) or electronic gas delivery system | 1 |  |  |  | 0.00 | -3.29 to 3.29 | 1.00 | Not applicable |
| COVID-19 | 2 |  |  |  | 0.00 | -3.29 to 3.29 | 1.00 | Not applicable |
| Without COVID-19 | 9 |  |  |  | -0.42 | -1.02 to 0.19 | 0.18 | 28% |
| Sedation up to 24 hours | 3 |  |  |  | -1.98 | -4.58 to 0.63 | 0.14 | 77% |
| Sedation more than 24 hours | 2 |  |  |  | 2.40 | -14.04 to 18.84 | 0.77 | Not applicable |
| Low risk of bias studies | 4 |  |  |  | -0.12 | -1.61 to 1.37 | 0.88 | 0% |
| **Length of ICU stay, hour** | 15 |  |  |  | 1.01 | -4.89 to 6.92 | 0.74 | 77% |
| Sevoflurane | 11 |  |  |  | 0.01 | -8.52 to 8.55 | 1.00 | 71% |
| Isoflurane | 3 |  |  |  | 70.37 | -30.69 to 171.42 | 0.17 | 73% |
| Either sevoflurane or isoflurane | 1 |  |  |  | 1.70 | -0.10 to 3.50 | 0.06 | Not applicable |
| Propofol as comparator | 12 |  |  |  | -0.87 | -5.84 to 4.09 | 0.73 | 72% |
| Surgical settings | 11 |  |  |  | -1.34 | -6.42 to 3.74 | 0.60 | 74% |
| Non-surgical settings | 4 |  |  |  | 17.92 | -55.72 to 91.55 | 0.63 | 52% |
| ARDS | 2 |  |  |  | 70.63 | 40.05 to 101.20 | <0.001 | 0% |
| Anesthetic conserving device  (dead space 100mL) | 10 |  |  |  | -0.27 | -3.21 to 2.66 | 0.86 | 29% |
| Anesthetic conserving device  (dead space 50mL) | 1 |  |  |  | 72.00 | 41.28 to 102.72 | <0.0001 | Not applicable |
| Electronic anesthetic delivery system | 1 |  |  |  | 9.60 | -4.56 to 23.76 | 0.18 | Not applicable |
| Either anesthetic conserving device (dead space 100mL) or electronic gas delivery system | 2 |  |  |  | -8.47 | -14.95 to -1.98 | 0.01 | 0% |
| Anesthetic vaporizer | 1 |  |  |  | 116.30 | 62.96 to 169.64 | <0.0001 | Not applicable |
| COVID-19 | 2 |  |  |  | 53.56 | -8.03 to 115.16 | 0.09 | 42% |
| Without COVID-19 | 13 |  |  |  | -1.46 | -6.48 to 3.55 | 0.57 | 70% |
| Sedation up to 24 hours | 3 |  |  |  | -4.54 | -12.31 to 3.24 | 0.25 | 57% |
| Sedation more than 24 hours | 5 |  |  |  | 54.67 | -2.21 to 111.55 | 0.06 | 68% |
| Low risk of bias studies | 7 |  |  |  | 18.49 | -10.45 to 47.43 | 0.21 | 82% |
| **Hypotension** | 10 | 128/531 | 134/561 | 1.01 |  | 0.75 to 1.36 | 0.95 | 43% |
| Sevoflurane | 7 | 65/254 | 76/269 | 0.81 |  | 0.48 to 1.36 | 0.42 | 60% |
| Isoflurane | 1 | 3/150 | 3/151 | 1.01 |  | 0.21 to 4.91 | 0.99 | Not applicable |
| Either sevoflurane or isoflurane | 2 | 60/127 | 55/141 | 1.01 |  | 0.75 to 1.36 | 0.17 | 0% |
| Propofol as comparator | 10 | 128/531 | 134/561 | 1.01 |  | 0.75 to 1.36 | 0.95 | 43% |
| Surgical settings | 8 | 120/484 | 114/501 | 1.11 |  | 0.83 to 1.47 | 0.49 | 35% |
| Non-surgical settings | 2 | 8/47 | 20/60 | 0.47 |  | 0.10 to 2.24 | 0.34 | 58% |
| ARDS | 1 | 1/28 | 7/32 | 0.16 |  | 0.02 to 1.25 | 0.08 | Not applicable |
| Anesthetic conserving device  (dead space 100mL) | 7 | 97/414 | 101/443 | 1.10 |  | 0.88 to 1.37 | 0.40 | 0% |
| Electronic anesthetic delivery system | 1 | 26/42 | 15/39 | 1.61 |  | 1.01 to 2.56 | 0.04 | Not applicable |
| Either anesthetic conserving device (dead space 100mL) or electronic gas delivery system | 2 | 5/75 | 18/79 | 0.31 |  | 0.12 to 0.79 | 0.01 | 0% |
| COVID-19 | 1 | 1/28 | 7/32 | 0.16 |  | 0.02 to 1.25 | 0.08 | Not applicable |
| Without COVID-19 | 9 | 127/503 | 127/529 | 1.07 |  | 0.83 to 1.39 | 0.60 | 33% |
| Sedation up to 24 hours | 1 | 21/49 | 19/50 | 1.13 |  | 0.70 to 1.82 | 0.62 | Not applicable |
| Sedation more than 24 hours | 2 | 10/169 | 16/179 | 0.83 |  | 0.43 to 1.58 | 0.56 | 0% |
| Low risk of bias studies | 4 | 35/272 | 30/273 | 1.00 |  | 0.41 to 2.43 | 0.99 | 57% |
| **Atrial fibrillation** | 7 | 67/749 | 59/757 | 1.15 |  | 0.74 to 1.79 | 0.53 | 31% |
| Sevoflurane | 4 | 48/472 | 49/465 | 0.93 |  | 0.56 to 1.54 | 0.77 | 36% |
| Isoflurane | 1 | 5/150 | 4/151 | 1.26 |  | 0.34 to 4.60 | 0.73 | Not applicable |
| Either sevoflurane or isoflurane | 2 | 14/127 | 6/141 | 2.59 |  | 1.03 to 6.54 | 0.53 | 31% |
| Propofol as comparator | 7 | 67/749 | 59/757 | 1.15 |  | 0.74 to 1.79 | 0.53 | 31% |
| Surgical settings | 6 | 40/403 | 36/416 | 1.19 |  | 0.64 to 2.19 | 0.58 | 43% |
| Non-surgical settings | 1 | 27/346 | 23/341 | 1.16 |  | 0.68 to 1.98 | 0.59 | Not applicable |
| ARDS | 1 | 27/346 | 23/341 | 1.16 |  | 0.68 to 1.98 | 0.59 | Not applicable |
| Anesthetic conserving device  (dead space 100mL) | 5 | 30/361 | 30/377 | 1.13 |  | 0.52 to 2.42 | 0.76 | 49% |
| Anesthetic conserving device (dead space 50mL) | 1 | 27/346 | 23/341 | 1.16 |  | 0.68 to 1.98 | 0.59 | Not applicable |
| Electronic anesthetic delivery system | 1 | 10/42 | 6/39 | 1.55 |  | 0.62 to 3.86 | 0.35 | Not applicable |
| COVID-19 | 1 | 27/346 | 23/341 | 1.16 |  | 0.68 to 1.98 | 0.59 | Not applicable |
| Without COVID-19 | 6 | 40/403 | 36/416 | 1.19 |  | 0.64 to 2.19 | 0.58 | 43% |
| Sedation up to 24 hours | 2 | 11/84 | 20/85 | 0.58 |  | 0.32 to 1.07 | 0.08 | 0% |
| Sedation more than 24 hours | 2 | 32/496 | 27/492 | 1.17 |  | 0.71 to 1.92 | 0.53 | 0% |
| Low risk of bias studies | 3 | 42/538 | 33/531 | 1.25 |  | 0.81 to 1.93 | 0.32 | 0% |
| **Acute kidney injury** | 8 | 284/728 | 237/704 | 1.14 |  | 0.67 to 1.93 | 0.62 | 60% |
| Sevoflurane | 6 | 269/537 | 216/534 | 1.36 |  | 0.80 to 2.32 | 0.26 | 48% |
| Isoflurane | 2 | 15/191 | 21/170 | 0.74 |  | 0.16 to 3.53 | 0.71 | 72% |
| Propofol as comparator | 7 | 276/687 | 235/685 | 1.09 |  | 0.61 to 1.95 | 0.76 | 65% |
| Surgical settings | 6 | 37/354 | 40/331 | 0.88 |  | 0.42 to 1.86 | 0.74 | 52% |
| Non-surgical settings | 2 | 247/374 | 197/373 | 2.36 |  | 0.46 to 12.10 | 0.30 | 82% |
| ARDS | 2 | 247/374 | 197/373 | 2.36 |  | 0.46 to 12.10 | 0.30 | 82% |
| Anesthetic conserving device  (dead space 100mL) | 4 | 17/265 | 22/245 | 0.83 |  | 0.29 to 2.40 | 0.73 | 37% |
| Anesthetic conserving device (dead space 50mL) | 1 | 236/346 | 195/341 | 1.19 |  | 1.06 to 1.34 | 0.003 | Not applicable |
| Electronic anesthetic delivery system | 1 | 16/42 | 9/39 | 1.65 |  | 0.83 to 3.29 | 0.15 | Not applicable |
| Either anesthetic conserving device (dead space 100mL) or electronic gas delivery system | 2 | 15/75 | 11/79 | 1.61 |  | 0.12 to 22.11 | 0.72 | 88% |
| COVID-19 | 2 | 247/374 | 197/373 | 2.36 |  | 0.46 to 12.10 | 0.30 | 82% |
| Without COVID-19 | 6 | 37/354 | 40/331 | 0.88 |  | 0.42 to 1.86 | 0.74 | 52% |
| Sedation up to 24 hours | 1 | 1/35 | 1/35 | 1.00 |  | 0.07 to 15.36 | 1.00 | Not applicable |
| Sedation more than 24 hours | 3 | 251/537 | 216/511 | 0.89 |  | 0.37 to 2.14 | 0.79 | 75% |
| Low risk of bias studies | 6 | 272/665 | 234/637 | 0.96 |  | 0.57 to 1.63 | 0.89 | 60% |
| **Delirium** | 7 | 43/357 | 42/356 | 1.04 |  | 0.72 to 1.50 | 0.85 | 0% |
| Sevoflurane | 4 | 25/174 | 27/175 | 1.00 |  | 0.63 to 1.60 | 0.99 | 0% |
| Isoflurane | 3 | 18/183 | 15/181 | 1.10 |  | 0.60 to 2.00 | 0.77 | 0% |
| Propofol as comparator | 5 | 33/324 | 32/326 | 1.03 |  | 0.67 to 1.57 | 0.89 | 0% |
| Surgical settings | 5 | 27/311 | 27/306 | 0.95 |  | 0.58 to 1.54 | 0.82 | 0% |
| Non-surgical settings | 2 | 16/46 | 15/50 | 1.17 |  | 0.67 to 2.06 | 0.58 | 0% |
| ARDS | 1 | 12/28 | 12/32 | 1.14 |  | 0.62 to 2.12 | 0.67 | Not applicable |
| Anesthetic conserving device  (dead space 100mL) | 5 | 27/282 | 27/277 | 0.95 |  | 0.59 to 1.54 | 0.84 | 0% |
| Either anesthetic conserving device (dead space 100mL) or electronic gas delivery system | 2 | 16/75 | 15/79 | 1.17 |  | 0.66 to 2.07 | 0.59 | 0% |
| COVID-19 | 1 | 12/28 | 12/32 | 1.14 |  | 0.62 to 2.12 | 0.67 | Not applicable |
| Without COVID-19 | 6 | 31/329 | 30/324 | 0.98 |  | 0.62 to 1.56 | 0.94 | 0% |
| Sedation up to 24 hours | 2 | 9/99 | 12/96 | 0.73 |  | 0.32 to 1.65 | 0.45 | 0% |
| Sedation more than 24 hours | 2 | 14/165 | 12/163 | 1.04 |  | 0.53 to 2.04 | 0.90 | 0% |
| Low risk of bias studies | 3 | 18/212 | 15/210 | 1.09 |  | 0.59 to 2.00 | 0.78 | 0% |
| **Postoperative nausea and vomiting** | 8 | 39/478 | 31/465 | 1.29 |  | 0.82 to 2.01 | 0.27 | 0% |
| Sevoflurane | 4 | 23/195 | 20/193 | 1.16 |  | 0.66 to 2.02 | 0.61 | 0% |
| Isoflurane | 2 | 5/191 | 3/170 | 1.34 |  | 0.32 to 5.60 | 0.69 | 0% |
| Desflurane | 1 | 0/25 | 2/28 | 0.22 |  | 0.01 to 4.44 | 0.33 | Not applicable |
| Either sevoflurane or isoflurane | 1 | 11/67 | 6/74 | 2.02 |  | 0.79 to 5.17 | 0.14 | Not applicable |
| Propofol as comparator | 7 | 38/437 | 30/446 | 1.32 |  | 0.84 to 2.09 | 0.23 | 0% |
| Surgical settings | 8 | 39/478 | 31/465 | 0.27 |  | 0.82 to 2.01 | 0.27 | 0% |
| Anesthetic conserving device  (dead space 100mL) | 6 | 38/406 | 28/390 | 1.35 |  | 0.85 to 2.14 | 0.20 | 0% |
| Either anesthetic conserving device (dead space 100mL) or electronic gas delivery system | 1 | 1/47 | 1/47 | 1.00 |  | 0.06 to 15.52 | 1.00 | Not applicable |
| Anesthetic vaporizer | 1 | 0/25 | 2/28 | 0.22 |  | 0.01 to 4.44 | 0.33 | Not applicable |
| Without COVID-19 | 8 | 39/478 | 31/465 | 1.29 |  | 0.82 to 2.01 | 0.27 | 0% |
| Sedation up to 24 hours | 4 | 22/173 | 21/174 | 1.10 |  | 0.63 to 1.92 | 0.74 | 0% |
| Sedation more than 24 hours | 2 | 5/191 | 3/170 | 1.34 |  | 0.32 to 5.60 | 0.69 | 0% |
| Low risk of bias studies | 3 | 6/238 | 4/217 | 1.26 |  | 0.35 to 4.48 | 0.72 | 0% |
| **Time to extubation from termination of sedation, minute** | 13 |  |  |  | -90.62 | -124.64 to -56.60 | <0.0001 | 90% |
| Sevoflurane | 6 |  |  |  | -94.57 | -144.60 to -44.53 | <0.001 | 88% |
| Isoflurane | 5 |  |  |  | -301.85 | -563.34 to -40.35 | 0.02 | 91% |
| Desflurane | 1 |  |  |  | -24.20 | -33.24 to -15.16 | <0.001 | Not applicable |
| Either sevoflurane or isoflurane | 1 |  |  |  | -115.00 | -156.76 to -73.24 | <0.001 | Not applicable |
| Propofol as comparator | 9 |  |  |  | -66.77 | -96.16 to -37.38 | <0.001 | 89% |
| Surgical settings | 11 |  |  |  | -71.50 | -103.47 to -39.52 | <0.001 | 89% |
| Non-surgical settings | 2 |  |  |  | -311.75 | -491.75 to -131.75 | <0.001 | 55% |
| Anesthetic conserving device  (dead space 100mL) | 8 |  |  |  | -124.93 | -189.51 to -60.34 | <0.001 | 89% |
| Electronic anesthetic delivery system | 1 |  |  |  | -59.30 | -91.45 to -27.15 | <0.001 | Not applicable |
| Either anesthetic conserving device (dead space 100mL) or electronic gas delivery system | 1 |  |  |  | -76.00 | -100.68 to -51.32 | <0.0001 | Not applicable |
| Anesthetic vaporizer | 3 |  |  |  | -408.92 | -834.42 to 16.58 | 0.06 | 93% |
| Without COVID-19 | 13 |  |  |  | -90.62 | -124.64 to -56.60 | <0.0001 | 90% |
| Sedation up to 24 hours | 4 |  |  |  | -62.83 | -100.48 to -25.17 | 0.001 | 89% |
| Sedation more than 24 hours | 5 |  |  |  | -355.23 | -642.20 to -68.26 | 0.02 | 92% |
| Low risk of bias studies | 5 |  |  |  | -54.65 | -110.08 to 0.79 | 0.05 | 83% |
| ARDS, acute respiratory distress syndrome; ICU, intensive care unit; COVID-19, coronavirus disease 2019 | | | | | | | | |

# **Table S5. Summary of sensitivity analyses for secondary outcomes**

| **Secondary outcome** | **No. of studies** | **Volatile sedation** | **Intravenous sedation** | **Relative risk** | **Mean difference** | **95% CI** | **P value** | **I^2^** |
| --- | --- | --- | --- | --- | --- | --- | --- | --- |
| **Duration of mechanical ventilation, hour** | 8 |  |  |  | -1.46 | -4.92 to 2.00 | 0.41 | 53% |
| Sevoflurane | 6 |  |  |  | -1.51 | -5.24 to 2.23 | 0.43 | 65% |
| Isoflurane | 2 |  |  |  | 19.63 | -36.93 to 76.20 | 0.50 | 0% |
| Propofol as comparator | 6 |  |  |  | -1.49 | 1.02 to 1.36 | 0.03 | 0% |
| Surgical settings | 5 |  |  |  | -1.46 | -3.89 to 0.65 | 0.16 | 39% |
| Non-surgical settings | 3 |  |  |  | 10.87 | -44.07 to 65.80 | 0.70 | 75% |
| ARDS | 2 |  |  |  | -18.12 | -199.74 to 163.50 | 0.84 | 71% |
| Low risk of bias studies | 5 |  |  |  | 35.74 | 3.96 to 67.52 | 0.03 | 0% |
| **Length of hospital stay, day** | 11 |  |  |  | -0.37 | -0.93 to 0.18 | 0.18 | 19% |
| Sevoflurane | 8 |  |  |  | -0.86 | -1.93 to 0.20 | 0.11 | 40% |
| Isoflurane | 1 |  |  |  | 19.63 | -36.93 to 76.20 | 0.50 | Not applicable |
| Either sevoflurane or isoflurane | 2 |  |  |  | 0.19 | -0.71 to 0.33 | 0.47 | 0% |
| Propofol as comparator | 10 |  |  |  | -0.40 | -0.99 to 0.19 | 0.18 | 28% |
| Surgical settings | 9 |  |  |  | -0.42 | -1.02 to 0.19 | 0.18 | 28% |
| Non-surgical settings | 2 |  |  |  | 0.00 | -3.29 to 3.29 | 1.00 | Not applicable |
| ARDS | 2 |  |  |  | 0.00 | -3.29 to 3.29 | 1.00 | Not applicable |
| Low risk of bias studies | 4 |  |  |  | -0.12 | -1.61 to 1.37 | 0.88 | 0% |
| **Length of ICU stay, hour** | 15 |  |  |  | 1.01 | -4.89 to 6.92 | 0.74 | 77% |
| Sevoflurane | 11 |  |  |  | 0.01 | -8.52 to 8.55 | 1.00 | 71% |
| Isoflurane | 3 |  |  |  | 70.37 | -30.69 to 171.42 | 0.17 | 73% |
| Either sevoflurane or isoflurane | 1 |  |  |  | 1.70 | -0.10 to 3.50 | 0.06 | Not applicable |
| Propofol as comparator | 12 |  |  |  | -0.87 | -5.84 to 4.09 | 0.73 | 72% |
| Surgical settings | 11 |  |  |  | -1.34 | -6.42 to 3.74 | 0.60 | 74% |
| Non-surgical settings | 4 |  |  |  | 17.92 | -55.72 to 91.55 | 0.63 | 52% |
| ARDS | 2 |  |  |  | 70.63 | 40.05 to 101.20 | <0.001 | 0% |
| Low risk of bias studies | 7 |  |  |  | 18.49 | -10.45 to 47.43 | 0.21 | 82% |
| **Hypotension** | 10 | 128/531 | 134/561 | 1.01 |  | 0.75 to 1.36 | 0.95 | 43% |
| Sevoflurane | 7 | 65/254 | 76/269 | 0.81 |  | 0.48 to 1.36 | 0.42 | 60% |
| Isoflurane | 1 | 3/150 | 3/151 | 1.01 |  | 0.21 to 4.91 | 0.99 | Not applicable |
| Either sevoflurane or isoflurane | 2 | 60/127 | 55/141 | 1.01 |  | 0.75 to 1.36 | 0.17 | 0% |
| Propofol as comparator | 10 | 128/531 | 134/561 | 1.01 |  | 0.75 to 1.36 | 0.95 | 43% |
| Surgical settings | 8 | 120/484 | 114/501 | 1.11 |  | 0.83 to 1.47 | 0.49 | 35% |
| Non-surgical settings | 2 | 8/47 | 20/60 | 0.47 |  | 0.10 to 2.24 | 0.34 | 58% |
| ARDS | 1 | 1/28 | 7/32 | 0.16 |  | 0.02 to 1.25 | 0.08 | Not applicable |
| Low risk of bias studies | 4 | 35/272 | 30/273 | 1.00 |  | 0.41 to 2.43 | 0.99 | 57% |
| **Atrial fibrillation** | 7 | 67/749 | 59/757 | 1.15 |  | 0.74 to 1.79 | 0.53 | 31% |
| Sevoflurane | 4 | 48/472 | 49/465 | 0.93 |  | 0.56 to 1.54 | 0.77 | 36% |
| Isoflurane | 1 | 5/150 | 4/151 | 1.26 |  | 0.34 to 4.60 | 0.73 | Not applicable |
| Either sevoflurane or isoflurane | 2 | 14/127 | 6/141 | 2.59 |  | 1.03 to 6.54 | 0.53 | 31% |
| Propofol as comparator | 7 | 67/749 | 59/757 | 1.15 |  | 0.74 to 1.79 | 0.53 | 31% |
| Surgical settings | 6 | 40/403 | 36/416 | 1.19 |  | 0.64 to 2.19 | 0.58 | 43% |
| Non-surgical settings | 1 | 27/346 | 23/341 | 1.16 |  | 0.68 to 1.98 | 0.59 | Not applicable |
| ARDS | 1 | 27/346 | 23/341 | 1.16 |  | 0.68 to 1.98 | 0.59 | Not applicable |
| Low risk of bias studies | 3 | 42/538 | 33/531 | 1.25 |  | 0.81 to 1.93 | 0.32 | 0% |
| **Acute kidney injury** | 8 | 284/728 | 237/704 | 1.14 |  | 0.67 to 1.93 | 0.62 | 60% |
| Sevoflurane | 6 | 269/537 | 216/534 | 1.36 |  | 0.80 to 2.32 | 0.26 | 48% |
| Isoflurane | 2 | 15/191 | 21/170 | 0.74 |  | 0.16 to 3.53 | 0.71 | 72% |
| Propofol as comparator | 7 | 276/687 | 235/685 | 1.09 |  | 0.61 to 1.95 | 0.76 | 65% |
| Surgical settings | 6 | 37/354 | 40/331 | 0.88 |  | 0.42 to 1.86 | 0.74 | 52% |
| Non-surgical settings | 2 | 247/374 | 197/373 | 2.36 |  | 0.46 to 12.10 | 0.30 | 82% |
| ARDS | 2 | 247/374 | 197/373 | 2.36 |  | 0.46 to 12.10 | 0.30 | 82% |
| Low risk of bias studies | 6 | 272/665 | 234/637 | 0.96 |  | 0.57 to 1.63 | 0.89 | 60% |
| **Delirium** | 7 | 43/357 | 42/356 | 1.04 |  | 0.72 to 1.50 | 0.85 | 0% |
| Sevoflurane | 4 | 25/174 | 27/175 | 1.00 |  | 0.63 to 1.60 | 0.99 | 0% |
| Isoflurane | 3 | 18/183 | 15/181 | 1.10 |  | 0.60 to 2.00 | 0.77 | 0% |
| Propofol as comparator | 5 | 33/324 | 32/326 | 1.03 |  | 0.67 to 1.57 | 0.89 | 0% |
| Surgical settings | 5 | 27/311 | 27/306 | 0.95 |  | 0.58 to 1.54 | 0.82 | 0% |
| Non-surgical settings | 2 | 16/46 | 15/50 | 1.17 |  | 0.67 to 2.06 | 0.58 | 0% |
| ARDS | 1 | 12/28 | 12/32 | 1.14 |  | 0.62 to 2.12 | 0.67 | Not applicable |
| Low risk of bias studies | 3 | 18/212 | 15/210 | 1.09 |  | 0.59 to 2.00 | 0.78 | 0% |
| **Postoperative nausea and vomiting** | 8 | 39/478 | 31/465 | 1.29 |  | 0.82 to 2.01 | 0.27 | 0% |
| Sevoflurane | 4 | 23/195 | 20/193 | 1.16 |  | 0.66 to 2.02 | 0.61 | 0% |
| Isoflurane | 2 | 5/191 | 3/170 | 1.34 |  | 0.32 to 5.60 | 0.69 | 0% |
| Desflurane | 1 | 0/25 | 2/28 | 0.22 |  | 0.01 to 4.44 | 0.33 | Not applicable |
| Either sevoflurane or isoflurane | 1 | 11/67 | 6/74 | 2.02 |  | 0.79 to 5.17 | 0.14 | Not applicable |
| Propofol as comparator | 7 | 38/437 | 30/446 | 1.32 |  | 0.84 to 2.09 | 0.23 | 0% |
| Surgical settings | 8 | 39/478 | 31/465 | 0.27 |  | 0.82 to 2.01 | 0.27 | 0% |
| Low risk of bias studies | 3 | 6/238 | 4/217 | 1.26 |  | 0.35 to 4.48 | 0.72 | 0% |
| **Time to extubation from termination of sedation, minute** | 13 |  |  |  | -90.62 | -124.64 to -56.60 | <0.0001 | 90% |
| Sevoflurane | 6 |  |  |  | -94.57 | -144.60 to -44.53 | <0.001 | 88% |
| Isoflurane | 5 |  |  |  | -301.85 | -563.34 to -40.35 | 0.02 | 91% |
| Desflurane | 1 |  |  |  | -24.20 | -33.24 to -15.16 | <0.001 | Not applicable |
| Either sevoflurane or isoflurane | 1 |  |  |  | -115.00 | -156.76 to -73.24 | <0.001 | Not applicable |
| Propofol as comparator | 9 |  |  |  | -66.77 | -96.16 to -37.38 | <0.001 | 89% |
| Surgical settings | 11 |  |  |  | -71.50 | -103.47 to -39.52 | <0.001 | 89% |
| Non-surgical settings | 2 |  |  |  | -311.75 | -491.75 to -131.75 | <0.001 | 55% |
| Low risk of bias studies | 5 |  |  |  | -54.65 | -110.08 to 0.79 | 0.05 | 83% |
| ARDS, acute respiratory distress syndrome; ICU, intensive care unit | | | | | | | | |

# **Table S6. Summary of additional sensitivity analyses for secondary outcomes**

| **Secondary outcome** | **No. of studies** | **Volatile sedation** | **Intravenous sedation** | **Relative risk** | **Mean difference** | **95% CI** | **P value** | **I^2^** |
| --- | --- | --- | --- | --- | --- | --- | --- | --- |
| **Duration of mechanical ventilation, hour** | 8 |  |  |  | -1.46 | -4.92 to 2.00 | 0.41 | 53% |
| Anesthetic conserving device | 8 |  |  |  | -1.46 | -4.92 to 2.00 | 0.41 | 53% |
| COVID-19 | 1 |  |  |  | 52.80 | 10.84 to 94.76 | 0.01 | NA |
| Without COVID-19 | 7 |  |  |  | -1.65 | -3.85 to 0.55 | 0.14 | 30% |
| Sedation up to 24 hours | 2 |  |  |  | -1.79 | -4.70 to 1.12 | 0.23 | 82% |
| Sedation more than 24 hours | 4 |  |  |  | 18.43 | -14.68 to 51.54 | 0.40 | 53% |
| **Length of hospital stay, day** | 11 |  |  |  | -0.37 | -0.93 to 0.18 | 0.18 | 19% |
| Anesthetic conserving device | 9 |  |  |  | -0.52 | -1.23 to 0.19 | 0.15 | 36% |
| Electronic anesthetic delivery system | 1 |  |  |  | 0.00 | -1.53 to 1.53 | 1.00 | NA |
| COVID-19 | 2 |  |  |  | 0.00 | -3.29 to 3.29 | 1.00 | NA |
| Without COVID-19 | 9 |  |  |  | -0.42 | -1.02 to 0.19 | 0.18 | 28% |
| Sedation up to 24 hours | 3 |  |  |  | -1.98 | -4.58 to 0.63 | 0.14 | 77% |
| Sedation more than 24 hours | 2 |  |  |  | 2.40 | -14.04 to 18.84 | 0.77 | NA |
| **Length of ICU stay, hour** | 15 |  |  |  | 1.01 | -4.89 to 6.92 | 0.74 | 77% |
| Anesthetic conserving device | 11 |  |  |  | -0.01 | -5.74 to 5.71 | 1.00 | 70% |
| Electronic anesthetic delivery system | 1 |  |  |  | 9.60 | -4.56 to 23.76 | 0.18 | NA |
| Anesthetic vaporizer | 1 |  |  |  | 116.30 | 62.96 to 169.64 | <0.0001 | NA |
| COVID-19 | 2 |  |  |  | 53.56 | -8.03 to 115.16 | 0.09 | 42% |
| Without COVID-19 | 13 |  |  |  | -1.46 | -6.48 to 3.55 | 0.57 | 70% |
| Sedation up to 24 hours | 3 |  |  |  | -4.54 | -12.31 to 3.24 | 0.25 | 57% |
| Sedation more than 24 hours | 5 |  |  |  | 54.67 | -2.21 to 111.55 | 0.06 | 68% |
| **Hypotension** | 10 | 128/531 | 134/561 | 1.01 |  | 0.75 to 1.36 | 0.95 | 43% |
| Anesthetic conserving device | 7 | 97/414 | 101/443 | 1.10 |  | 0.88 to 1.37 | 0.40 | 0% |
| Electronic anesthetic delivery system | 1 | 26/42 | 15/39 | 1.61 |  | 1.01 to 2.56 | 0.04 | NA |
| COVID-19 | 1 | 1/28 | 7/32 | 0.16 |  | 0.02 to 1.25 | 0.08 | NA |
| Without COVID-19 | 9 | 127/503 | 127/529 | 1.07 |  | 0.83 to 1.39 | 0.60 | 33% |
| Sedation up to 24 hours | 1 | 21/49 | 19/50 | 1.13 |  | 0.70 to 1.82 | 0.62 | NA |
| Sedation more than 24 hours | 2 | 10/169 | 16/179 | 0.83 |  | 0.43 to 1.58 | 0.56 | 0% |
| **Atrial fibrillation** | 7 | 67/749 | 59/757 | 1.15 |  | 0.74 to 1.79 | 0.53 | 31% |
| Anesthetic conserving device | 6 | 57/707 | 53/718 | 1.10 |  | 0.66 to 1.85 | 0.71 | 39% |
| Electronic anesthetic delivery system | 1 | 10/42 | 6/39 | 1.55 |  | 0.62 to 3.86 | 0.35 | NA |
| COVID-19 | 1 | 27/346 | 23/341 | 1.16 |  | 0.68 to 1.98 | 0.59 | NA |
| Without COVID-19 | 6 | 40/403 | 36/416 | 1.19 |  | 0.64 to 2.19 | 0.58 | 43% |
| Sedation up to 24 hours | 2 | 11/84 | 20/85 | 0.58 |  | 0.32 to 1.07 | 0.08 | 0% |
| Sedation more than 24 hours | 2 | 32/496 | 27/492 | 1.17 |  | 0.71 to 1.92 | 0.53 | 0% |
| **Acute kidney injury** | 8 | 284/728 | 237/704 | 1.14 |  | 0.67 to 1.93 | 0.62 | 60% |
| Anesthetic conserving device | 5 | 253/611 | 217/586 | 0.95 |  | 0.47 to 1.92 | 0.89 | 52% |
| Electronic anesthetic delivery system | 1 | 16/42 | 9/39 | 1.65 |  | 0.83 to 3.29 | 0.15 | NA |
| COVID-19 | 2 | 247/374 | 197/373 | 2.36 |  | 0.46 to 12.10 | 0.30 | 82% |
| Without COVID-19 | 6 | 37/354 | 40/331 | 0.88 |  | 0.42 to 1.86 | 0.74 | 52% |
| Sedation up to 24 hours | 1 | 1/35 | 1/35 | 1.00 |  | 0.07 to 15.36 | 1.00 | NA |
| Sedation more than 24 hours | 3 | 251/537 | 216/511 | 0.89 |  | 0.37 to 2.14 | 0.79 | 75% |
| **Delirium** | 7 | 43/357 | 42/356 | 1.04 |  | 0.72 to 1.50 | 0.85 | 0% |
| Anesthetic conserving device | 5 | 27/282 | 27/277 | 0.95 |  | 0.59 to 1.54 | 0.84 | 0% |
| COVID-19 | 1 | 12/28 | 12/32 | 1.14 |  | 0.62 to 2.12 | 0.67 | NA |
| Without COVID-19 | 6 | 31/329 | 30/324 | 0.98 |  | 0.62 to 1.56 | 0.94 | 0% |
| Sedation up to 24 hours | 2 | 9/99 | 12/96 | 0.73 |  | 0.32 to 1.65 | 0.45 | 0% |
| Sedation more than 24 hours | 2 | 14/165 | 12/163 | 1.04 |  | 0.53 to 2.04 | 0.90 | 0% |
| **Postoperative nausea and vomiting** | 8 | 39/478 | 31/465 | 1.29 |  | 0.82 to 2.01 | 0.27 | 0% |
| Anesthetic conserving device | 6 | 38/406 | 28/390 | 1.35 |  | 0.85 to 2.14 | 0.20 | 0% |
| Anesthetic vaporizer | 1 | 0/25 | 2/28 | 0.22 |  | 0.01 to 4.44 | 0.33 | NA |
| Without COVID-19 | 8 | 39/478 | 31/465 | 1.29 |  | 0.82 to 2.01 | 0.27 | 0% |
| Sedation up to 24 hours | 4 | 22/173 | 21/174 | 1.10 |  | 0.63 to 1.92 | 0.74 | 0% |
| Sedation more than 24 hours | 2 | 5/191 | 3/170 | 1.34 |  | 0.32 to 5.60 | 0.69 | 0% |
| **Time to extubation from termination of sedation, minute** | 13 |  |  |  | -90.62 | -124.64 to -56.60 | <0.0001 | 90% |
| Anesthetic conserving device | 8 |  |  |  | -124.93 | -189.51 to -60.34 | <0.001 | 89% |
| Electronic anesthetic delivery system | 1 |  |  |  | -59.30 | -91.45 to -27.15 | <0.001 | NA |
| Anesthetic vaporizer | 3 |  |  |  | -408.92 | -834.42 to 16.58 | 0.06 | 93% |
| Without COVID-19 | 13 |  |  |  | -90.62 | -124.64 to -56.60 | <0.0001 | 90% |
| Sedation up to 24 hours | 4 |  |  |  | -62.83 | -100.48 to -25.17 | 0.001 | 89% |
| Sedation more than 24 hours | 5 |  |  |  | -355.23 | -642.20 to -68.26 | 0.02 | 92% |
| COVID-19, coronavirus disease 2019 | | | | | | | | |

COVID-19, coronavirus 2019; NA, not applicable

# **Table S7. Exploratory physiological outcomes**

|  |  |  | **PaCO_2_, mean (SD), mmHg** | | | | **pH, mean (SD)** | | | |
| --- | --- | --- | --- | --- | --- | --- | --- | --- | --- | --- |
|  |  |  | **Volatile** | | **IV** | | **Volatile** | | **IV** | |
| **First author,**  **year** | **Device** | **Dead space,**  **mL** | **Baseline** | **Day 2** | **Baseline** | **Day 2** | **Baseline** | **Day 2** | **Baseline** | **Day 2** |
| Jabaudon M,  2017 | ACD | 100 | 43.7 (7.4) | 43.8 (6.6) | 45.0 (7.5) | 41.6 (5.0) | 7.32 (0.11) | 7.38 (0.08) | 7.37 (0.09) | 7.42 (0.06) |
| Meiser A,  2021 | ACD | 50 or  100 | 43.3 (9.3) | 47.2 (9.9) | 43.5 (11.1) | 45.9 (8.1) | 7.4 (0.1) | 7.4 (0.1) | 7.4 (0.1) | 7.4 (0.1) |
| Martínez-Castro S,  2023 | ACD | 100 | 45.4 (14.2) | 59.1 (15.5) | 48.5 (15.2) | 44.5 (7.8) | 7.37 (0.14) | 7.31 (0.11) | 7.35 (0.10) | 7.42 (0.04) |
| Jabaudon M,  2025 | ACD | 50 | 42.7 (8.9) | 46.0 (8.9) | 43.7 (8.9) | 41.3 (6.7) | 7.36 (0.09) | 7.38 (0.08) | 7.35 (0.10) | 7.41 (0.08) |
| ACD, anesthetic conserving device; IV, intravenous | | | | | | | | | | |

# **Changes from the original review protocol**

*October 21, 2024*

We initially planned to include both randomized controlled trials (RCTs) and propensity score-matched observational studies. However, a meta-analysis pooling the results from randomized controlled trials and non-randomized observational studies typically introduces heterogeneity in study design and confounding, which is discouraged by the Cochrane collaborative. Thus, we decided to include only RCTs while excluding observational studies.

*March 19, 2025*

On March 18, 2025, a large, multicenter RCT (SESAR trial) enrolling 700 patients was published. This study was the first RCT adequately powered to evaluate the effects of volatile sedation on clinically relevant outcomes, such as mortality and duration of mechanical ventilation. Including this study will allow us to evaluate such patient-centered outcomes in this systematic review and meta-analysis. Thus, we decided to re-perform the literature review to include this study and provide the most updated and comprehensive randomized evidence.

*April 24, 2025*

We selected mortality, rather than time to extubation, as the primary outcome. Initially, due to the limited number and small sample sizes of available trials, we focused on time to extubation as a surrogate for arousal following sedation withdrawal. However, the recent publication of a large-scale trial provided sufficient statistical power to assess the effects on more clinically relevant outcomes, including mortality. Moreover, the SESAR trial reported a potential increase in mortality associated with volatile sedation, raising safety concerns and further underscoring the need to investigate mortality as a primary endpoint.

# **Supplementary references**

1. Spencer EM, Willatts SM, Prys-Roberts C. Plasma inorganic fluoride concentrations during and after prolonged (greater than 24 h) isoflurane sedation: effect on renal function. Anesth Analg. 1991;73:731–7.

2. Millane TA, Bennett ED, Grounds RM. Isoflurane and propofol for long-term sedation in the intensive care unit. A crossover study. Anaesthesia. 1992;47:768–74.

3. Gómez JI, Tamayo E, Cortejoso J, Cóbreces MJ. [Isoflurane versus propofol for sedation after heart surgery]. Rev Esp Anestesiol Reanim. 1995;42:261–8.

4. Tanigami H, Yahagi N, Kumon K, Watanabe Y, Haruna M, Matsui J, et al. Long-term sedation with isoflurane in postoperative intensive care in cardiac surgery. Artif Organs. 1997;21:21–3.

5. Freye E, Sundermann S, Wilder-Smith OH. No inhibition of gastro-intestinal propulsion after propofol- or propofol/ketamine-N2O/O2 anaesthesia. A comparison of gastro-caecal transit after isoflurane anaesthesia. Acta Anaesthesiol Scand. 1998;42:664–9.

6. Hellström J, Öwall A, Bergström J, Sackey PV. Cardiac outcome after sevoflurane versus propofol sedation following coronary bypass surgery: a pilot study. Acta Anaesthesiol Scand. 2011;55:460–7.

7. Villa F, Iacca C, Molinari AF, Giussani C, Aletti G, Pesenti A, et al. Inhalation versus endovenous sedation in subarachnoid hemorrhage patients: effects on regional cerebral blood flow. Crit Care Med. 2012;40:2797–804.

8. Steurer MP, Steurer MA, Baulig W, Piegeler T, Schläpfer M, Spahn DR, et al. Late pharmacologic conditioning with volatile anesthetics after cardiac surgery. Crit Care. 2012;16:R191.

9. Soukup J, Selle A, Wienke A, Steighardt J, Wagner N-M, Kellner P. Efficiency and safety of inhalative sedation with sevoflurane in comparison to an intravenous sedation concept with propofol in intensive care patients: study protocol for a randomized controlled trial. Trials. 2012;13:135.

10. Bösel J, Purrucker JC, Nowak F, Renzland J, Schiller P, Pérez EB, et al. Volatile isoflurane sedation in cerebrovascular intensive care patients using AnaConDa(®): effects on cerebral oxygenation, circulation, and pressure. Intensive Care Med. 2012;38:1955–64.

11. Guerrero Orriach JL, Galán Ortega M, Ramirez Aliaga M, Iglesias P, Rubio Navarro M, Cruz Mañas J. Prolonged sevoflurane administration in the off-pump coronary artery bypass graft surgery: beneficial effects. J Crit Care. 2013;28:879.e13-18.

12. Wong K, Wasowicz M, Ferguson N, Grewal D, Doherty M, Steel A. SCAVENGING OF VOLATILE ANESTHETICS DURING LONG-TERM SEDATION OF CRITICAL CARE PATIENTS. Can J Anesth/J Can Anesth. 2014;61:1–165.

13. Ramirez M, Guerrero-Orriach J, Galan M, Iglesias P, Rubio M, Bellido I, et al. Cardioprotective ef fect of sevoflurane vs. propofol during anaesthesia and postoperative period in of f-pump coronary artery bypass graf t surgery. European Journal of Anaesthesiology. 2014;Abstracts Programme.

14. Jerath A, Ferguson ND, Steel A, Wijeysundera D, Macdonald J, Wasowicz M. The use of volatile anesthetic agents for long-term critical care sedation (VALTS): study protocol for a pilot randomized controlled trial. Trials. 2015;16:560.

15. Bonvini JM, Beck-Schimmer B, Kuhn SJ, Graber SM, Neff TA, Schläpfer M. Late Post-Conditioning with Sevoflurane after Cardiac Surgery--Are Surrogate Markers Associated with Clinical Outcome? PLoS One. 2015;10:e0132165.

16. Bellgardt M, Bomberg H, Herzog-Niescery J, Dasch B, Vogelsang H, Weber TP, et al. Survival after long-term isoflurane sedation as opposed to intravenous sedation in critically ill surgical patients: Retrospective analysis. Eur J Anaesthesiol. 2016;33:6–13.

17. Hassan WMNW, Nasir YM, Zaini RHM, Shukeri WFWM. Target-controlled Infusion Propofol Versus Sevoflurane Anaesthesia for Emergency Traumatic Brain Surgery: Comparison of the Outcomes. Malays J Med Sci. 2017;24:73–82.

18. Staudacher DL, Hamilton S-K, Duerschmied D, Biever PM, Zehender M, Bode C, et al. Isoflurane or propofol sedation in patients with targeted temperature management after cardiopulmonary resuscitation: A single center study. J Crit Care. 2018;45:40–4.

19. Meiser A, Groesdonk HV, Bonnekessel S, Volk T, Bomberg H. Inhalation Sedation in Subjects With ARDS Undergoing Continuous Lateral Rotational Therapy. Respir Care. 2018;63:441–7.

20. Walczak KD, Castro VO, Grewal D, Jerath A, Wasowicz M, Ferguson ND, et al. Impact of Volatile Anesthetics for Long-Term Sedation in Critically Ill Patients on Cognitive Impairment at 3-Months Follow-Up.

21. Türktan M, Güleç E, Hatipoğlu Z, Ilgınel MT, Özcengiz D. The Effect of Sevoflurane and Dexmedetomidine on Pulmonary Mechanics in ICU Patients. Turk J Anaesthesiol Reanim. 2019;47:206–12.

22. Donadello K, Simari S, Bonora E, Citino M, Cogo G, Schweiger V, et al. What is the perfect place for sevoflurane in our ICUs? ICMx. 2019;7:55, s40635-019-0265-y.

23. Sung T-Y, Lee D-K, Bang J, Choi J, Shin S, Kim T-Y. Remifentanil-based propofol-supplemented vs. balanced sevoflurane-sufentanil anesthesia regimens on bispectral index recovery after cardiac surgery: a randomized controlled study. Anesth Pain Med (Seoul). 2020;15:424–33.

24. Jung S, Na S, Kim HB, Joo HJ, Kim J. Inhalation sedation for postoperative patients in the intensive care unit: initial sevoflurane concentration and comparison of opioid use with propofol sedation. Acute Crit Care. 2020;35:197–204.

25. Bailly P, Egreteau P-Y, Ehrmann S, Thille AW, Guitton C, Grillet G, et al. Inased (inhaled sedation in ICU) trial protocol: a multicentre randomised open-label trial. BMJ Open. 2021;11:e042284.

26. Müller-Wirtz LM, Behne F, Kermad A, Wagenpfeil G, Schroeder M, Sessler DI, et al. Isoflurane promotes early spontaneous breathing in ventilated intensive care patients: A post hoc subgroup analysis of a randomized trial. Acta Anaesthesiol Scand. 2022;66:354–64.

27. Jabaudon M, Zhai R, Blondonnet R, Bonda WLM. Inhaled sedation in the intensive care unit. Anaesthesia Critical Care & Pain Medicine. 2022;41:101133.

28. Flinspach AN, Herrmann E, Raimann FJ, Zacharowski K, Adam EH. Evaluation of volatile sedation in the postoperative intensive care of patients recovering from heart valve surgery: protocol for a randomised, controlled, monocentre trial. BMJ Open. 2022;12:e057804.

29. Becher T, Meiser A, Guenther U, Bellgardt M, Wallenborn J, Kogelmann K, et al. Isoflurane vs. propofol for sedation in invasively ventilated patients with acute hypoxemic respiratory failure: an a priori hypothesis substudy of a randomized controlled trial. Ann Intensive Care. 2022;12:116.

30. Bracht H, Meiser A, Wallenborn J, Guenther U, Kogelmann KM, Faltlhauser A, et al. ICU- and ventilator-free days with isoflurane or propofol as a primary sedative - A post- hoc analysis of a randomized controlled trial. J Crit Care. 2023;78:154350.

31. Sunnybrook Health Sciences Centre. SedAting With Volatile Anesthetics Critically Ill COVID-19 Patients in ICU: Effects On Ventilatory Parameters And Survival. Multicentre Open-label, Pragmatic, Randomized Controlled Trial and a Parallel Prospective (Non-randomized) Cohort Study [Internet]. clinicaltrials.gov; 2023 Apr. Report No.: NCT04415060. Available from: https://clinicaltrials.gov/study/NCT04415060

32. Palacios-Chavarria A. A Randomized Pilot Clinical Trial of the Effects in Oxygenation and Hypoxic Pulmonary Vasoconstriction of Sevoflurane in Patient&#x27;s Whit ARDS Secondary to Severe Acute Respiratory Syndrome Coronavirus 2 (SARS-CoV2) [Internet]. clinicaltrials.gov; 2021 Aug. Report No.: NCT04998253. Available from: https://clinicaltrials.gov/study/NCT04998253

33. Flinspach AN. Prospective Evaluation of Volatile Sedation Management in Critical Care After Free Flap Surgery [Internet]. clinicaltrials.gov; 2023 Oct. Report No.: NCT05707884. Available from: https://clinicaltrials.gov/study/NCT05707884

34. Kong KL, Willatts SM, Prys-Roberts C. Isoflurane compared with midazolam for sedation in the intensive care unit. BMJ. 1989;298:1277–80.

35. Sackey PV, Martling C-R, Granath F, Radell PJ. Prolonged isoflurane sedation of intensive care unit patients with the Anesthetic Conserving Device. Crit Care Med. 2004;32:2241–6.

36. Sackey PV, Martling C-R, Carlswärd C, Sundin O, Radell PJ. Short- and long-term follow-up of intensive care unit patients after sedation with isoflurane and midazolam--a pilot study. Crit Care Med. 2008;36:801–6.

37. Beck-Schimmer B, Schadde E, Pietsch U, Filipovic M, Dübendorfer-Dalbert S, Fodor P, et al. Early sevoflurane sedation in severe COVID-19-related lung injury patients. A pilot randomized controlled trial. Ann Intensive Care. 2024;14:41.

38. Hellström J, Öwall A, Sackey PV. Wake-up times following sedation with sevoflurane versus propofol after cardiac surgery. Scand Cardiovasc J. 2012;46:262–8.

39. Jabaudon M, Boucher P, Imhoff E, Chabanne R, Faure J-S, Roszyk L, et al. Sevoflurane for Sedation in Acute Respiratory Distress Syndrome. A Randomized Controlled Pilot Study. Am J Respir Crit Care Med. 2017;195:792–800.

40. Guinot P-G, Ellouze O, Grosjean S, Berthoud V, Constandache T, Radhouani M, et al. Anaesthesia and ICU sedation with sevoflurane do not reduce myocardial injury in patients undergoing cardiac surgery: A randomized prospective study. Medicine (Baltimore). 2020;99:e23253.

41. Meiser A, Volk T, Wallenborn J, Guenther U, Becher T, Bracht H, et al. Inhaled isoflurane via the anaesthetic conserving device versus propofol for sedation of invasively ventilated patients in intensive care units in Germany and Slovenia: an open-label, phase 3, randomised controlled, non-inferiority trial. Lancet Respir Med. 2021;9:1231–40.

42. Martínez-Castro S, Monleón B, Puig J, Ferrer Gomez C, Quesada M, Pestaña D, et al. Sedation with Sevoflurane versus Propofol in COVID-19 Patients with Acute Respiratory Distress Syndrome: Results from a Randomized Clinical Trial. J Pers Med. 2023;13:925.

43. Jabaudon M, Quenot J-P, Badie J, Audard J, Jaber S, Rieu B, et al. Inhaled Sedation in Acute Respiratory Distress Syndrome. 2025;

44. Flinspach AN, Raimann FJ, Kaiser P, Pfaff M, Zacharowski K, Neef V, et al. Volatile versus propofol sedation after cardiac valve surgery: a single-center prospective randomized controlled trial. Critical Care. 2024;28:111.

45. Röhm KD, Wolf MW, Schöllhorn T, Schellhaass A, Boldt J, Piper SN. Short-term sevoflurane sedation using the Anaesthetic Conserving Device after cardiothoracic surgery. Intensive Care Med. 2008;34:1683–9.

46. Röhm KD, Mengistu A, Boldt J, Mayer J, Beck G, Piper SN. Renal integrity in sevoflurane sedation in the intensive care unit with the anesthetic-conserving device: a comparison with intravenous propofol sedation. Anesth Analg. 2009;108:1848–54.

47. Soro M, Gallego L, Silva V, Ballester MT, Lloréns J, Alvariño A, et al. Cardioprotective effect of sevoflurane and propofol during anaesthesia and the postoperative period in coronary bypass graft surgery: a double-blind randomised study. Eur J Anaesthesiol. 2012;29:561–9.

48. Wąsowicz M, Jerath A, Luksun W, Sharma V, Mitsakakis N, Meineri M, et al. Comparison of propofol-based versus volatile-based anaesthesia and postoperative sedation in cardiac surgical patients: a prospective, randomized, study. Anaesthesiol Intensive Ther. 2018;50:200–9.

49. Soukup J, Michel P, Christel A, Schittek GA, Wagner N-M, Kellner P. Prolonged sedation with sevoflurane in comparison to intravenous sedation in critically ill patients - A randomized controlled trial. J Crit Care. 2023;74:154251.

50. Jerath A, Wong K, Wasowicz M, Fowler T, Steel A, Grewal D, et al. Use of Inhaled Volatile Anesthetics for Longer Term Critical Care Sedation: A Pilot Randomized Controlled Trial. Critical Care Explorations. 2020;2:e0281.
